# Selective induction of cancer cell death by VDAC1‐based peptides and their potential use in cancer therapy

**DOI:** 10.1002/1878-0261.12313

**Published:** 2018-05-19

**Authors:** Anna Shteinfer‐Kuzmine, Zohar Amsalem, Tasleem Arif, Alexandra Zooravlov, Varda Shoshan‐Barmatz

**Affiliations:** ^1^ Department of Life Sciences National Institute for Biotechnology in the Negev Ben‐Gurion University of the Negev Beer‐Sheva Israel

**Keywords:** apoptosis, cancer, metabolism, mitochondria, peptides, VDAC1

## Abstract

Mitochondrial VDAC1 mediates cross talk between the mitochondria and other parts of the cell by transporting anions, cations, ATP, Ca^2+^, and metabolites and serves as a key player in apoptosis. As such, VDAC1 is involved in two important hallmarks of cancer development, namely energy and metabolic reprograming and apoptotic cell death evasion. We previously developed cell‐penetrating VDAC1‐derived peptides that interact with hexokinase (HK), Bcl‐2, and Bcl‐xL to prevent the anti‐apoptotic activities of these proteins and induce cancer cell death, with a focus on leukemia and glioblastoma. In this study, we demonstrated the sensitivity of a panel of genetically characterized cancer cell lines, differing in origin and carried mutations, to VDAC1‐based peptide‐induced apoptosis. Noncancerous cell lines were less affected by the peptides. Furthermore, we constructed additional VDAC1‐based peptides with the aim of improving targeting, selectivity, and cellular stability, including R‐Tf‐D‐LP4, containing the transferrin receptor internalization sequence (Tf) that allows targeting of the peptide to cancer cells, known to overexpress the transferrin receptor. The mode of action of the VDAC1‐based peptides involves HK detachment, interfering with the action of anti‐apoptotic proteins, and thus activating multiple routes leading to an impairment of cell energy and metabolism homeostasis and the induction of apoptosis. Finally, in xenograft glioblastoma, lung, and breast cancer mouse models, R‐Tf‐D‐LP4 inhibited tumor growth while inducing massive cancer cell death, including of cancer stem cells. Thus, VDAC1‐based peptides offer an innovative new conceptual framework for cancer therapy.

**Abbreviations**
CSC: cancer stem cell
Cyto *c*: cytochrome *c*
HK: hexokinase
IHC: immunohistochemistry
OMM: outer mitochondrial membrane
VDAC1: voltage‐dependent anion channel 1

## 1 Introduction

Cancer cells share a common set of hallmarks, including unlimited proliferation potential, self‐sufficiency in terms of growth signals, reprogramed metabolism, and avoiding apoptosis by alterations in the expression levels of pro‐ and anti‐apoptotic proteins, as well as due to reduced caspase function and impaired death receptor signaling (Antonsson and Martinou, 2000). Overexpression of anti‐apoptotic proteins, such as Bcl‐2 and Bcl‐xL, has been demonstrated in numerous cancers, including colon, thyroid, breast, and endometrial cancer. Moreover, Bcl‐2 expression is correlated with the degree of aggressiveness and resistance to chemotherapy‐induced apoptosis (Llambi and Green, 2011).

Mitochondria occupy a central position in cell life and death, with mitochondrial bioenergetics, biosynthesis, and signaling being critical for tumorigenesis. Multistep mitochondria‐mediated apoptosis that relies on the intrinsic pathway can be initiated by a variety of stimuli involving outer mitochondrial membrane (OMM) permeabilization, leading to the release of apoptotic proteins, such as cytochrome *c* (Cyto *c*) and apoptosis‐inducing factor (AIF). This results in the activation of a cascade of cysteine proteases (caspases), subsequently leading to organized cell demise (Kroemer *et al*., 2007).

It is now recognized that the OMM protein voltage‐dependent anion channel 1 (VDAC1) is a key player in mitochondria‐mediated apoptosis (Keinan *et al*., 2010; Lemasters and Holmuhamedov, 2006; Shimizu *et al*., 2001; Shoshan‐Barmatz *et al*., 2010, 2017b). VDAC1 participates in the release of apoptotic proteins and regulates apoptosis through interactions with Bcl‐2 family proteins and hexokinase (HK) (Keinan *et al*., 2010; Lemasters and Holmuhamedov, 2006; Shimizu *et al*., 2001; Shoshan‐Barmatz *et al*., 2010, 2017b). Indeed, accumulated findings indicate that both anti‐ and pro‐apoptotic proteins, including Bax, Bcl‐2, and Bcl‐xL, interact with VDAC1 to regulate mitochondria‐mediated apoptosis (Abu‐Hamad *et al*., 2009; Arbel and Shoshan‐Barmatz, 2010; Arbel *et al*., 2012; Malia and Wagner, 2007; Shi *et al*., 2003; Shimizu *et al*., 1999, 2000; Shoshan‐Barmatz *et al*., 2009, 2017a; Sugiyama *et al*., 2002; Tajeddine *et al*., 2008; Tsujimoto, 2003). HK, Bcl‐2, and Bcl‐xL, moreover, interact with bilayer‐reconstituted VDAC1 to reduce the channel conductance of wild‐type but not of certain mutated forms of VDAC1, including N‐terminally truncated VDAC1 (Arbel and Shoshan‐Barmatz, 2010; Arbel *et al*., 2012; Shimizu *et al*., 1999; Shoshan‐Barmatz *et al*., 2009; Sugiyama *et al*., 2002; Tsujimoto, 2003). Similarly, HK, Bcl‐2, and Bcl‐xL prevent apoptosis induced by various stimuli in cells expressing wild‐type but not mutated VDAC1 (Abu‐Hamad *et al*., 2009; Arbel and Shoshan‐Barmatz, 2010; Arbel *et al*., 2012). It was also proposed that VDAC1 interacts with both Bax and Bcl‐xL to form a tertiary complex (Shi *et al*., 2003). Indeed, a direct interaction between Bcl‐xL and VDAC1 was demonstrated by NMR (Malia and Wagner, 2007).

In addition to its role in apoptosis, VDAC1 plays a crucial role in regulating the metabolic and energetic functions of the cell. VDAC1 assumes a crucial position at the OMM, serving as the main interface between mitochondrial and cellular metabolisms, mediating the transport of anions, cations including Ca^2+^, ATP, and many metabolites between the mitochondria and the cytosol (Shoshan‐Barmatz *et al*., 2010). Moreover, its location at the boundary between the mitochondria and the cytosol enables VDAC1 to function as an anchoring site for a diverse set of cytosolic proteins, such as HK, tubulin, actin, and several dozen other proteins (Shoshan‐Barmatz *et al*., 2017c), that mediate metabolic and signaling cross talk between cytosol and mitochondria and regulate the integration of mitochondrial functions with other cellular activities (Shoshan‐Barmatz *et al*., 2010, 2017b).

The ability of cancer cells to evade chemotherapy‐induced apoptosis is a major cause of treatment failure and aggressive tumorigenesis. Overexpression of anti‐apoptotic proteins, such as Bcl‐2 and Bcl‐xL (Llambi and Green, 2011), and downregulation of apoptosis‐promoting factors, such as Bax and Fas (McCurrach *et al*., 1997), have been detected in many primary tumors and tumor cell lines, with such alterations having been linked to chemotherapeutic resistance.

At the same time, a small population of cells within a tumor, known as cancer stem cells (CSCs), are resistant to traditional cancer treatment (Weiswald *et al*., 2015; Zhang *et al*., 2017). The CSCs hypothesis postulates that a subpopulation of malignant cells constantly supplies the tumor with cancerous cells. These CSCs possess unique markers, a gene expression profile like that of embryonic and somatic stem cells, self‐renewal, and multipotent differentiation abilities (Frank *et al*., 2010; Lee and Herlyn, 2007). However, in contrast to normal stem cells, CSCs are not regulated, such that their continuous expansion allows them to maintain a tumor. CSCs are, moreover, associated with drug resistance and cancer recurrence. CSCs have been identified in a number of solid tumors, such as breast, lung, and colon cancers, brain tumors, and melanoma (Dawood *et al*., 2014).

Although selective induction of cancer cell death remains a major concern and a challenge for the development of new anticancer therapies, treatments designed to activate the apoptotic machinery and abolish the ability of tumors to evade apoptosis are still considered to be most effective (Fulda *et al*., 2010).

Rapidly growing tumor cells typically display altered aerobic glycolysis (the Warburg effect), with this metabolic reprograming being directly related to tumor growth and progression (Vander Heiden *et al*., 2009). The Warburg effect can be described as a metabolic phenotype characterized by enhanced glycolysis and suppression of mitochondrial metabolism at any level of oxygen. However, although enhanced glycolysis is a prominent feature of most tumor cells, cancer cells in fact use both glycolysis and oxidative phosphorylation (OXPHOS), with the degree each strategy is used being dependent on the prevailing normoxic or hypoxic environmental conditions and the capacity of such cells to express adequate levels of oncogenes and tumor suppressor gene products for cell growth (Majeed *et al*., 2012). In addition, rapidly growing tumors meet their metabolic demands by increased expression of genes encoding glucose transporters and glycolytic enzymes, including HK. As a key mediator of aerobic glycolysis, HK catalyzes the first and rate‐limiting step in glucose metabolism, that is, the conversion of glucose to glucose 6‐phosphate (G‐6‐P). HK thus lies at the apex of the glycolytic pathway that provides the metabolic intermediates required by those biosynthetic pathways on which a transformed cell makes heavy demand (Mathupala *et al*., 2010; Pedersen, 2007). By binding to VDAC1 at the OMM, HK gains direct access to mitochondrially produced ATP. The interaction of HK with VDAC1 protects against apoptosis, with this protection being abolishing upon dissociation of the HK‐VDAC1 complex (Azoulay‐Zohar *et al*., 2004; Pastorino *et al*., 2002; Shoshan‐Barmatz *et al*., 2017a). Indeed, disrupting the VDAC1‐HK interaction is a potential strategy for cancer treatment (Cao *et al*., 2008; Fulda *et al*., 2010; Galluzzi *et al*., 2008; Goldin *et al*., 2008). In addition, dissociation of HK from VDAC1 regulates Bax translocation to the mitochondria (Pastorino *et al*., 2002).

In our previous studies, key sequences and amino acid residues of VDAC1 required for Bcl‐2, Bcl‐xL, and HK binding and protection against cell death were identified (Abu‐Hamad *et al*., 2009; Arbel and Shoshan‐Barmatz, 2010; Arzoine *et al*., 2009; Shi *et al*., 2003; Shoshan‐Barmatz *et al*., 2009). On the basis of these findings, we designed and tested 27 versions of VDAC1‐based cell‐penetrating peptides representing two different structural regions of VDAC1 to identify the most stable short apoptosis‐inducing peptides (Prezma *et al*., 2013). These cell‐penetrating peptides included the D‐Δ(1‐14)N‐Ter‐Antp peptide, composed of the VDAC1 N‐terminal region (15–26 amino acids) fused to Antp (Penetrating), a 16‐residue‐long sequence from the *Drosophila* Antennapedia homeodomain, both containing amino acids in the D‐configuration, and Tf‐D‐LP4, comprising a VDAC1‐derived cytosol‐facing loop sequence, defined as LP4, fused to a human transferrin receptor (hTfR)‐recognition sequence, HAIYPRH (Tf) (Daniels *et al*., 2012), with only the amino acids of the VDAC1‐derived sequence being in D‐configuration. hTfR is highly expressed in many cancers (Daniels *et al*., 2012). Additional versions of these VDAC1‐based peptides are presented in this study. These VDAC1‐based peptides serve as a decoy, targeting the anti‐apoptotic proteins, and thereby inhibiting the protection against apoptosis. As such, they induce cell death in cancerous but not in noncancerous cells (Abu‐Hamad *et al*., 2008, 2009; Arbel and Shoshan‐Barmatz, 2010; Arbel *et al*., 2012; Arzoine *et al*., 2009; Shi *et al*., 2003; Shoshan‐Barmatz *et al*., 2009; Zaid *et al*., 2005).

In this work, we demonstrate that VDAC1‐based peptides are able to selectively induce apoptotic cell death in a large panel of cancer cell lines, regardless of their cancer origin or the mutations carried, including mutated p53. Furthermore, we explored the mode of action of the peptides and report on the triple mode of action, namely impairing cell energy homeostasis, prevention of the protective effects of anti‐apoptotic proteins, and induction of massive apoptosis. Finally, in subcutaneous glioblastoma (U‐87MG), lung cancer (A549), and breast cancer (MDA‐MB‐231) cell‐derived xenograft mouse models, a VDAC1‐based peptide inhibited tumor development by inducing massive apoptotic cell death involving enhanced expression of pro‐apoptotic proteins, reversal of the metabolic reprograming of the tumor cells, and elimination of cancer stem cells (CSCs).

## 2 Materials and methods

### 2.1 Materials

Adenosine triphosphate (ATP), carbonyl cyanide‐p‐trifluoromethoxyphenylhydrazone (FCCP), cytochalasin B, 4′,6‐diamidino‐2‐phenylindole (DAPI), dimethyl sulfoxide (DMSO), glucose, glucose‐6‐phosphate dehydrogenase (G6PDH), leupeptin, beta‐mercaptoethanol, phenylmethylsulfonyl fluoride (PMSF), propidium iodide (PI), sucrose, Tris, and trypan blue were purchased from Sigma (St. Louis, MO, USA). Dithiothreitol (DTT) was purchased from Thermo Fisher Scientific (Waltham, MA, USA). HEPES was purchased from Holland Moran (Fisher Scientific, Geel, Belgium). The cell transfection agent jetPRIME was from PolyPlus (Illkirch, France). Nicotinamide adenine dinucleotide phosphate (NADP) was purchased from Bio Basic Canada (Markham, ON, Canada). Annexin V (FITC) was obtained from Alexis Biochemicals (Lausen, Switzerland). Dulbecco's modified Eagle's medium (DMEM), BSA 7.5%, MEM nonessential amino acids (NEAA) and B27 and N2 supplements, phosphate‐buffered saline (PBS), and Roswell Park Memorial Institute medium (RPMI) were purchased from Gibco (Grand Island, NY, USA). DMEM/HAMS‐F12, Hanks’ balanced salts solution (HBSS) without calcium, and the supplements fetal bovine serum (FBS) and penicillin/streptomycin were purchased from Biological Industries (Beit‐Haemek, Israel). TUNEL and CellTiter‐Glo Luminescent Cell Viability Assay kits were obtained from Promega (Madison, WI, USA). 3,3‐Diaminobenzidine (DAB) was obtained from ImmPact‐DAB (Burlingame, CA, USA). Primary and secondary antibodies used in immunoblotting and immunohistochemistry (IHC), as well as their dilutions, are listed in Table S2.

### 2.2 Cell lines and culture

Forty‐one established cancer cell lines were used in this study: U‐251MG, U‐118MG, U‐87MG, and LN‐18 (human glioblastoma); GL‐261 (mouse glioblastoma); SH‐SY5Y (human neuroblastoma); N2A (mouse neuroblastoma); CNS‐1 and C6 (rat glioma); PANC1, AsPC1, and T3M4 (human pancreas carcinoma); PANC2 and SVR (mouse pancreas); HepG2 and HuH‐7 (human hepatocellular carcinoma); DU‐145 and PC‐3 (human prostate carcinoma); A‐375, CRL‐1619, HTB‐66, and HTB‐72 (human melanoma); B16F10, B16F10.9, K1735 16, and K1735 M4 (mouse melanoma); MEC‐1, MO1043, and CLL (human B‐cell chronic lymphocytic leukemia); Molt‐4 (human acute lymphoblastic leukemia); K‐562 (human acute myelocytic leukemia); U‐937 (human histiocytic leukemia); Jurkat (human T‐cell leukemia); THP‐1 (human acute monocytic leukemia); MDA‐MB‐231 and MCF‐7 (human breast adenocarcinoma); TSA (mouse breast cancer); A549 (human lung adenocarcinoma); H358 (human bronchoalveolar carcinoma); SKOV‐3 (human ovarian carcinoma); and HeLa (human cervix adenocarcinoma). Five nonmalignant cell lines were used in the study: PWR‐1E (human prostate epithelial cells), T‐Rex‐293 (human transformed primary embryonic kidney fibroblasts), MDCK (dog kidney epithelial), PBCs (mouse primary brain cells), and PBMCs (human peripheral blood mononuclear cells). For additional cell line properties, see Tables 1 and 3.

**Table 1:** Cell death induction by the Antp‐LP4 and N‐Ter‐Antp peptides in various cancer cell lines. Summary of the half‐maximal cell death activity (EC_50_) values (μm) for cell death induction by the N‐Ter‐Antp and Antp‐LP4 peptides in various cancer cell lines (calculated from the maximal extent of cell death obtained). Cell lines were incubated with different peptide concentrations for 90 min (for cells in suspension; Nos. 1–6), or 6 h (for adherent cells; Nos. 7–24). Cell death was determined by PI staining and FACS analysis. Data represent mean ± SE (*n* = 3). Mutation data taken from COSMIC (http://www.sanger.ac.uk/genetics/CGP/CellLines/)

No	Cell line	Origin	Mutation status	Peptide EC_50_, μm	
				N‐Ter‐Antp	Antp‐LP4
Group I					
1	K‐562	Human acute myelocytic leukemia	TP53, CBL, ASXL1, EPAS1, PDGFRA	1.9 ± 1.0	1.2 ± 0.1
2	U‐937	Human histiocytic leukemia	TP53, PTEN	1.9 ± 0.5	1.9 ± 0.7
3	Jurkat	Human T‐cell leukemia	TP53, PTEN, STAT3, LCK, FAT1	1.2 ± 0.9	1.3 ± 0.5
4	Molt‐4	Human acute lymphoblastic leukemia	TP53, PTEN, MTOR, NRAS, HRAS	3.5 ± 0.5	3.4 ± 2.0
5	MEC‐1	Human B‐cell chronic lymphocytic leukemia	TP53, PTEN, CDKN2A, BRCA1/2	3.0 ± 0.3	2.4 ± 0.5
6	THP‐1	Human monocytic leukemia	TP53, CDKN2A/B NRAS, MLLT4	1.7 ± 0.1	1.3 ± 0.5
7	MDA‐MB‐231	Human breast adenocarcinoma	TP53, KRAS, BRAF, CDKN2A, PDGFRA	5.2 ± 1.0	8.1 ± 2.4
8	HuH‐7	Human hepatocellular carcinoma	TP53, CYLD, AMER1, PTPRC, UBR5	4.0 ± 1.0	2.3 ± 1.5
9	PC‐3	Human prostatic small cell carcinoma	TP53	0.9 ± 0.4	5.9 ± 1.5
10	DU‐145	Human prostate carcinoma	TP53, CDKN2A, BRCA1, RET, BCL10, ABL2	1.5 ± 0.4	4.0 ± 0.5
11	T3M4	Human pancreatic cancer	TP53, CDKN2A, SMAD	4.2 ± 0.9	2.0 ± 0.7
12	U‐87MG	Human glioblastoma	PTEN, CDKN2A, KRAS, NF1, PCM1	4.5 ± 1.8	6.2 ± 1.3
Group II					
13	CNS‐1	Rat glioma	NR	6.5 ± 1.4	1.3 ± 0.8
14	HeLa	Human cervix adenocarcinoma	AR, TPR	8.2 ± 2.3	3.9 ± 1.1
15	SKOV‐3	Human ovarian carcinoma	TP53, FLT3, APC, TP63, BCL11B	8.5 ± 2.3	6.9 ± 0.7
16	A‐375	Human malignant melanoma	BRAF, MTOR, RUNX1, MECOM, MYH9	10.2 ± 1.3	33.8 ± 3.2
17	A‐549	Human lung adenocarcinoma	KRAS, KEAP1, FLT3, ATR, CBL, STK11	8.3 ± 1.5	6.4 ± 1.1
18	H358	Human bronchoalveolar carcinoma	TP53, KRAS, CEBPA	9.8 ± 0.8	7.6 ± 2.5
19	PWR‐1E	Human prostate epithelial noncancer cells	NUP98	7.8 ± 2.5	15.5 ± 2.0
20	MCF‐7	Human breast adenocarcinoma	CDKN2A, BCR, ERBB4, FLT3, GATA3	8.9 ± 2.1	10.1 ± 0.2
21	HepG2	Human hepatocellular carcinoma	ΝΡΑS, ΧΤΝΝΒ1	9.8 ± 4.7	8.6 ± 3.0
Group III					
22	PANC1	Human pancreas epithelioid carcinoma	TP53, KRAS, CDKN2A	18.9 ± 1.7	28.7 ± 2
23	AsPC1	Human pancreas carcinoma	TP53, KRAS, SMAD4, FBXW7	19.8 ± 0.3	12.0 ± 7.3
24	T‐Rex‐293	Human primary embryonic kidney fibroblast	Nonmalignant	18.0 ± 2.8	29.3 ± 4.0

Cell lines were maintained at 37 °C and 5% CO_2_ in the recommended culture medium supplemented with 10% FBS, 1 mm l‐glutamine, 100 U·mL^−1^ penicillin, and 100 μg·mL^−1^ streptomycin. PWR‐1E cells were maintained in keratinocyte‐SFM (serum‐free medium; Invitrogen, Carlsbad, CA, USA).

### 2.3 Peptide synthesis and solution preparation

Custom VDAC1‐based peptides were synthesized by GL Biochem (Shanghai, China) to a level of > 85% purity. The peptides were dissolved in 5% or 20% dimethyl sulfoxide and stored in aliquots at −20 °C. Peptide concentrations were determined using absorbance at 280 nm and the specific molar excitation coefficient, as calculated based on amino acid composition. For all experiments, the final concentration of DMSO in control and peptide‐containing samples was ≤ 0.5%. The major peptides used in this study, with the cell‐penetrating sequence underlined, the amino acids added to form a loop‐shaped tryptophan zipper in italics, and amino acids in D‐configuration in bold, are listed as follows:

Antp‐LP4, RQIKIWFQNRRMKWKK *SWTWE*‐199‐KKLETAVNLAWTAGNSN‐216‐*KWTWK* (43 residues); Tf‐D‐LP4, HAIYPRH **S** ***WTWE***‐199‐**KKLETAVNLAWTAGNSN** ‐216‐ ***KWTWK*** (34 residues); Retro‐Tf‐D‐LP4 (R‐Tf‐D‐LP4), ***KWTWK***‐216‐**NSNGATWALNVATE LKK**‐199‐***EWTWS*** HRPYIAH (34 residues); N‐Ter‐Antp, 1‐MAVPPTYADLGKSARDVFTKGYGFGL‐26‐RQIKIWFQNRRMKWKK (42 residues); and D‐Δ(1‐14)N‐Ter‐Antp, 15‐**RDVF TKGYGFG**L‐26‐**RQIKIWFQNRRMKWKK** (28 residues). Other peptides are presented in Table S1.

### 2.4 Isolation of peripheral blood mononuclear cells (PBMCs)

PBMCs were isolated from the venous blood of healthy donors by Ficoll‐Paque PLUS density gradient centrifugation. After informed consent, venous blood (10 mL) was drawn from normal adult donors. Blood was collected into heparin tubes and was diluted 1 : 1 with balance solution composed of two stock solutions, A (1% d‐glucose, 50 mm CaCl_2_, 0.98 mm MgCl_2_, 5.4 mm KCl, and 0.145 m Tris/HCl, pH 7.6) and B (0.14 m NaCl) in a 1 : 9 ratio. The resulting mix was carefully layered onto Ficoll‐Paque PLUS (GE Healthcare, Uppsala, Sweden) (10 mL of diluted blood to 15 mL Ficoll) in 50‐mL conical tubes. The tubes were centrifuged at 400 ***g*** (with minimal acceleration and deceleration) at 18–20 °C for 40 min. The fine layer of mononuclear cells was transferred to a new centrifuge tube, washed three times with balance solution, and resuspended in culture medium appropriate to the application. Cell viability was analyzed by assaying trypan blue (0.25%) exclusion, as counted with a Countess automated cell counter (Invitrogen).

### 2.5 Determination of cellular ATP and Ca^2+^ levels

Cellular ATP levels were estimated using a luciferase‐based assay (CellTiter‐Glo; Promega). HeLa cells (3 × 10^5 ^mL^−1^) were incubated with the indicated concentrations of Tf‐D‐LP4 or R‐Tf‐D‐LP4 peptides for 3 h, washed twice with PBS, and transferred to 96‐well white plates at densities of 1 × 10^5^ cells·mL^−1^. ATP levels were assayed according to the manufacturer's protocol, and luminescence was recorded using an Infinite M1000 plate reader (Tecan, Männedorf, Switzerland).

Cytosolic Ca^2+^ levels [Ca^2+^]i were analyzed using Fluo‐4‐AM. A549 cells were harvested after the appropriate treatment, collected (1500 g for 10 min), washed with HBSS buffer (5.33 mm KCl, 0.44 mm KH_2_PO_4_, 138 mm NaCl, 4 mm NaHCO_3_, 0.3 mm Na_2_HPO_4_, 5.6 mm glucose, 0.03 mm phenol red) supplemented with 1.8 mm CaCl_2_ (HBSS+), and incubated with 2 μm Fluo‐4 in 200 μL HBSS(+) buffer in the dark for 30 min at 37 °C. After washing the remaining dye, [Ca^2+^]i was measured immediately by FACS and analyzed with an EC800 Flow cytometer Analyzer–Eclipse (Sony Biotechnology, San Jose, CA, USA).

### 2.6 Cell treatment with VDAC1‐based peptides and cell death analysis

Leukemia cells, U‐937, MEC‐1 cells (4 × 10^5^ or 8 × 10^5^ cells/sample, respectively), as well as other leukemia or suspension cell lines, were incubated in 200 μL serum‐free medium with various concentrations of the peptide for 90 min at 23–25 °C, collected by centrifugation (500 ***g***, 5 min), washed with PBS, and analyzed for cell death. Adherent cells (6 × 10^5 ^mL^−1^ at 70–80% confluence) were incubated with the peptide of interest in 500 μL serum‐free recommended culture medium for 3–6 h at 37 °C and 5% CO_2_. The cells were then trypsinized, centrifuged (1500 ***g***, 5 min), washed with PBS, and analyzed for the desired activity. Following peptide treatment, cells were subjected to cell death analysis. Propidium iodide (PI) staining was performed by addition of PI (6.25 μg·mL^−1^) to the cells and immediate analysis by the EC800 Flow cytometer Analyzer–Eclipse or the FACS caliber (Becton‐Dickinson, San Jose, CA, USA) and bd cellquest pro software (BD Biosciences, San Jose, CA, USA).

For PI and annexin V–FITC staining, A549 or MDA‐MB‐231 cells, untreated or treated with VDAC1‐based peptides, were collected (1500 g for 5 min), washed, and resuspended in 200 μL binding buffer (10 mm HEPES/NaOH (pH 7.4), 140 mm NaCl, and 2.5 mm CaCl_2_). Annexin V–FITC staining was performed according to the recommended protocol. Cells were then washed once with binding buffer and resuspended in 200 μL binding buffer, to which PI was added immediately before flow cytometric analysis. At least 10 000 events were collected, recorded on a dot plot, and analyzed by flow cytometer. Acridine orange/ethidium bromide staining was carried as described previously (Zaid *et al*., 2005), with cells visualized by fluorescence microscopy (Olympus LX2‐KSP, Tokyo, Japan), and images were captured with a CCD camera. About 200 cells were counted for each experiment.

### 2.7 Mitochondria‐bound HK detachment

Peptide‐induced HK detachment from mitochondria to the cytosol was analyzed by following the cellular localization of HK‐I‐GFP before and after incubation with Tf‐D‐LP4 or D‐∆(1‐14)N‐Ter‐Antp peptides. HeLa cells were grown on coverslips and transfected with plasmid pEGFP‐HK‐I. Twenty‐four hours post‐transfection, the cells were incubated for 3 h with a solution containing 0.07% DMSO or Tf‐D‐LP4 or D‐∆(1‐14)N‐Ter‐Antp peptide to the indicated final concentration, fixed for 15 min with 4% paraformaldehyde in PBS, rinsed with PBS, permeabilized with 0.3% Triton X‐100, and stained with DAPI. Cells imaging was carried out by confocal microscopy (Olympus 1X81).

Peptide‐induced HK detachment from mitochondria to the cytosol was also analyzed by immunoblotting. A549 or HeLa cells were incubated with Tf‐D‐LP4 or R‐Tf‐D‐LP4 peptide (3, 5, 10 μm for 3 h), harvested, washed with PBS and gently resuspended (6 mg·mL^−1^) in ice‐cold buffer (100 mm KCl, 2.5 mm MgCl_2_, 250 mm sucrose, 20 mm HEPES/KOH (pH 7.5), 0.2 mm EDTA, 1 μg·mL^−1^ leupeptin, 5 μg·mL^−1^ cytochalasin B, and 0.1 mm phenylmethylsulfonyl fluoride) containing 0.025% digitonin, and incubated for 10 min on ice. Samples were centrifuged at 12 000 ***g*** at 4 °C for 10 min, and the obtained supernatants (cytosolic fraction) and pellets (mitochondria) were analyzed by immunoblotting using anti‐HK‐I, anti‐VDAC1, and anti‐GAPDH antibodies, and then with secondary HRP‐conjugated antibodies.

### 2.8 Recombinant HK‐I and II expression, purification, and activity assay

Human HK‐I and HK‐II were cloned into plasmid pET‐His DNA and expressed in *Escherichia coli* BL21 upon IPTG induction. HK was purified from lysed cells using a HiTrap nickel column (GE Healthcare). Following dialysis, the imidazole‐eluted protein was further purified using a Blue HiTrap column (GE Healthcare). HK was eluted using 10 mm glucose, 20 mm Tris/HCl (pH 8.5), 20% glycerol, and 1.5 mm glucose‐6‐phosphate. Fractions were analyzed by SDS/PAGE and tested for HK activity. The purified active fractions were combined and stored in aliquots at −80 °C.

Purified HK‐I (13 μg·mL^−1^) was preincubated with Tf‐D‐LP4 or R‐Tf‐D‐LP4 peptides in reaction buffer comprising 20 mm HEPES/KOH (pH 7.8), 10 mm glucose, 4 mm MgCl_2_, and 0.6 mm NADP and assayed for HK activity. Change in absorbance at 340 nm (NADH production) was spectrophotometrically measured following addition of 1 mm ATP and 0.05 unit·mL^−1^ glucose‐6‐phosphate dehydrogenase.

### 2.9 Cytochrome *c* release assay

A549 cells were treated with the Tf‐D‐LP4, R‐Tf‐D‐LP4, or D‐∆N‐Ter‐Antp peptide (10 μm, 3 h), washed, paraformaldehyde‐fixed (4%, 15 min), permeabilized with 0.3% Triton X‐100 in PBS (5 min), incubated with anti‐Cyto *c* antibodies and then with secondary Alexa Fluor 488‐conjugated anti‐mouse antibodies, and stained with DAPI (1 : 2000). Samples were imaged by confocal microscopy (Olympus 1X81). Cyto *c* release was also analyzed by cell fractionation into cytosolic and mitochondrial fractions and immunoblotting using anti‐Cyto *c* antibodies.

### 2.10 Microscale thermophoresis (MST)

Purified HK‐I and HK‐II were fluorescently labeled using a NanoTemper BLUE protein‐labeling kit. Fluorescently labeled HK‐I and HK‐II (830 and 100 nm, respectively) were incubated with different concentrations of Antp‐LP4, Tf‐D‐LP4, or R‐Tf‐D‐LP4 (1–100 μm) in PBS. After 20 min of incubation, 3‐ to 5‐μL aliquots were loaded into MST‐grade glass capillaries (NanoTemper Technologies, Munich, Germany) and thermophoresis was measured with a NanoTemper Monolith‐NT115 system (40% light‐emitting diode, 40% IR laser power).

### 2.11 Cross‐linking experiments

HeLa cells were treated with the R‐Tf‐D‐LP4 peptide (5–15 μm, 5 h), harvested, washed with PBS (pH 8.3), and incubated for 15 min with the cross‐linking reagent EGS at a ratio of 3 mg protein·mL^−1^/300 μm EGS. Aliquots (60–80 μg protein) were subjected to SDS/PAGE and immunoblotting using anti‐VDAC1 antibodies. Analysis of immunoreactive VDAC1 monomers, dimers, and multimer bands was performed using fusion‐fx (Vilber Lourmat, Collégien, France).

### 2.12 Spheroid formation

Conditions for spheroid formation were as described previously (Pollard *et al*., 2009; Weiswald *et al*., 2015). Briefly, U‐87MG, A549, or MDA‐MB‐231 cells (10^5^) were cultured in 12‐well plates coated with a soft agar (5%) layer and treated with or without peptide in stem cell culture medium (Pollard *et al*., 2009) composed of DMEM/HAMS‐F12 supplemented with BSA (0.012%), glucose (8 mm), beta‐mercaptoethanol (0.05 mm), B27 (× 0.5), N_2_ (× 0.5), EGF (0.01 μg·mL^−1^), and FGF (0.01 μg·mL^−1^). Following a 24‐ and 48‐h incubation at 37 °C in a 5% CO_2_ atmosphere, the cultures were photographed.

### 2.13 Xenograft mouse model

U‐87MG cells (3 × 10^6^/mouse), A549 cells (5 × 10^6^/mouse), or MDA‐MB‐231 cells (3 × 10^6^/mouse) were inoculated subcutaneously (s.c.) into the hind leg flanks of athymic 8‐week‐old male nude mice (Envigo, Huntingdon, UK). Tumor size was measured using a digital caliper and volume was calculated, and when reached 50–100 mm^3^ for each tumor type, mice were randomly divided into two groups (five mice per group). One group was intratumorally injected with HBSS (5.33 mm KCl, 0.44 mm KH_2_PO_4_, 138 mm NaCl, 4 mm NaHCO_3_, 0.3 mm Na_2_HPO4, and 5.6 mm glucose, pH 7.3) containing 0.05% DMSO and the second group with the peptide in HBSS containing 0.05% DMSO, with the R‐Tf‐D‐LP4 peptide at a final concentration of 40 or 60 μm. The xenografts were injected (two points, 20 μL per tumor) every two days. Beginning on the day of inoculation, mouse weight and tumor volume were monitored twice a week. Experiments were terminated after 14, 29, or 20 days for U‐87MG‐, MDA‐MB‐231‐, or A549‐based tumors, respectively. The mice were sacrificed and tumors were excised. Half of each tumor was fixed in 4% buffered formaldehyde, paraffin‐embedded, and processed for IHC, while the second half was frozen in liquid nitrogen for immunoblotting and qPCR analyses. These experimental protocols were approved by the Institutional Animal Care and Use Committee of Ben‐Gurion University.

### 2.14 Gel electrophoresis and immunoblotting

Cells or tumor tissues were lysed using lysis buffer (50 mm Tris/HCl (pH 7.5), 150 mm NaCl, 1 mm EDTA, 1.5 mm MgCl_2_, 10% glycerol, and 1% Triton X‐100, supplemented with a protease inhibitor cocktail (Calbiochem, San Diego, CA, USA). The lysates were then centrifuged at 12 000 ***g*** (10 min at 4 °C), and aliquots (10–40 μg of protein) were subjected to SDS/PAGE and immunoblotting using various primary antibodies (sources and dilutions are provided in Table S2), followed by incubation with appropriate HRP‐conjugated secondary antibodies (i.e., anti‐mouse, anti‐rabbit, or anti‐goat). Blots were developed using enhanced chemiluminescence (Biological Industries). Band intensities were analyzed by densitometry using fusion‐fx (Vilber Lourmat) software, and the values were normalized to the intensities of the appropriate β‐actin signal that served as a loading control.

### 2.15 IHC of tumor tissue sections

Formalin‐fixed, paraffin‐embedded sections of control and peptide‐treated tumors (peptide‐TTs) derived from U‐87MG, A549, and MDA‐MB‐231 cells were hematoxylin–eosin‐stained and probed with appropriate antibodies for IHC staining. IHC staining was performed on 5‐μm‐thick formalin‐fixed and paraffin‐embedded tumor tissue sections. The tissue sections were deparaffinized by placing the slides at 60 °C for 1 h and using xylene, followed by rehydration with a graded ethanol series (100–50%). Antigen retrieval for some proteins (AIF, ATP synthase 5a, CD44, citrate synthase, Cyto *c*, cytochrome *c* oxidase subunit IVc, GAPDH, Glut‐1, KLF4, Nestin, p53, S100b, SOX2, VDAC1) was performed in 0.01 m citrate buffer (pH 6.0). For HK‐I and Ki‐67, antigen retrieval was performed in 10 mm Tris/EDTA (pH 9) or 0.5 m Tris/EDTA (pH 10) for 30 min at 95–98 °C. Sections were washed with PBS containing 0.1% Triton X‐100 (pH 7.4), and nonspecific antibody binding was reduced by incubating the sections in 10% normal goat serum for 2 h overnight at 4 °C with primary antibodies (sources and dilutions used detailed in Table S2) and washing with PBST. Endogenous peroxidase activity was blocked by incubating the sections in 3% H_2_O_2_ for 15 min. Sections were washed thoroughly with PBST, incubated with the appropriate secondary antibodies (Table S2) for 2 h, and washed five times in PBST, and the peroxidase reaction was subsequently visualized by incubating with 3,3‐diaminobenzidine (DAB) (ImmPact‐DAB). After rinsing in water, the sections were counterstained with hematoxylin and mounted with mounting medium. Finally, the sections were observed under a microscope (Leica DM2500, Allendale, NJ, USA) and images were collected at 20**×** magnification with the same light intensity and exposure time. Nonspecific control experiments were carried out using the same protocols but omitting incubation with the primary antibodies. Hematoxylin–eosin (H&E) staining was performed as described previously (Elsam, 2008).

### 2.16 TUNEL assay

Paraffin‐embedded‐fixed tumor sections were processed for a TUNEL assay using the DeadEnd Fluorometric TUNEL system according to the manufacturer's instructions. Sections were deparaffinized, equilibrated in PBS, permeabilized with proteinase K (20 μg·mL^−1^ in PBS), postfixed in 4% paraformaldehyde, and incubated in TdT reaction mix for 1 h at 37 °C in the dark. Slides were then washed in 2× saline–sodium citrate (SSC) buffer and counterstained with propidium iodide (1 μg·mL^−1^), and coverslipped with Vectashield mounting medium (Vector Laboratories, Burlingame, CA, USA). Fluorescent images of apoptotic cells (green) and cell nuclei (red) were captured using a confocal microscope (Olympus 1X81).

### 2.17 RNA preparation and quantitative real‐time PCR (qPCR)

Total RNA was isolated from untreated and peptide‐treated tumors (three mice each) using an RNeasy Mini Kit (Qiagen, Hilden, Germany) according to the manufacturer's instructions. Total RNA quality was analyzed using the Agilent RNA 6000 Nano Kit. The obtained samples’ RNA integrity values for total RNA were 8–10. qPCR was performed using specific primers (KiCqStart Primers; Sigma‐Aldrich) in triplicate, using Power SYBR Green Master Mix (Applied Biosystems, Foster City, CA, USA). The levels of target genes were normalized relative to β‐actin mRNA levels. Samples were amplified by a 7300 Real‐Time PCR System (Applied Biosystems) for denaturation step of 5 min at 95 °C and 40 cycles using the following PCR parameters: 95 °C for 15 s, 60 °C for 1 min, and 72 °C for 1 min. The copy numbers for each sample were calculated by the CT‐based calibrated standard curve method. The mean fold changes (±SE) of three replicates were calculated. The genes examined and primers used are listed in Table S3.

### 2.18 Statistics and data analysis

Means ± SE of results obtained from three independent experiments are presented. Comparisons were made nonparametrically by the Mann–Whitney *U*‐test. Statistical significance is reported at *P* < 0.05(*), *P* < 0.01(**), *P* < 0.001(***), or *P* < 0.0001(****).

## 3 Results

In a previous study (Prezma *et al*., 2013), over 27 versions of cell‐penetrating VDAC1‐based peptides were designed and screened for apoptosis induction in chronic lymphocytic leukemia (CLL)‐derived lymphocytes. Three of these peptides were tested *in vivo* in glioblastoma (Shteinfer‐Kuzmine *et al*., 2017). In this study, we further optimized these cell‐permeable VDAC1‐based peptides, testing them against a panel of cancer cell lines differing in their origin and mutational status so as to explore the mode of action of selected peptides. We validated the activity of one such peptide on tumors established from three different cancer types in mouse models, demonstrating that the peptide not only inhibits tumor growth but also reverses unique properties of the cancer cells.

### 3.1 VDAC1‐based peptides induce cancer cell death regardless of cancer type

**Figure 1:**
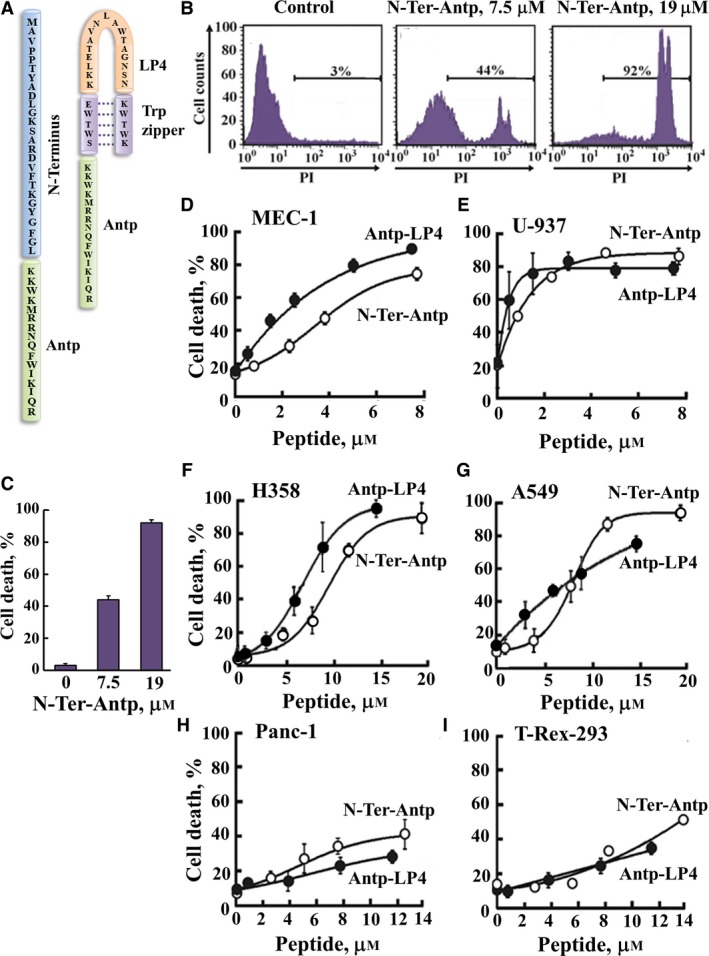
The VDAC1‐based peptides Antp‐LP4 and N‐Ter‐Antp induce dramatic cell death of several cancer cell lines. (A) Schematic illustration of the structure of N‐Ter‐Antp and the loop‐shaped Antp‐LP4 peptides. VDAC1‐derived sequences N terminus and LP4 are in blue and orange, respectively. The cell‐penetrating peptide (Antp) is in green, and the loop‐shaped stabilizing sequence, a tryptophan zipper (Trp zipper), is in purple. (B,C) A375 cells were incubated with the indicated concentrations of N‐Ter‐Antp peptide in the appropriate serum‐free growth medium for 6 h at 37 °C, and cell death was analyzed by PI staining and flow cytometry. (B) Representative FACS analysis for cells treated with or without (control) the indicated concentrations of the N‐Ter‐Antp peptide. (C) Quantitative analysis of FACS experiments. Data represent mean values ± SE (*n* = 3). (D,E) MEC‐1 and U‐937 cells were incubated with the indicated concentrations of the Antp‐LP4 (●) or N‐Ter‐Antp (○) peptides in the appropriate serum‐free growth medium for 90 min at 23–25 °C, and cell death was analyzed by PI staining and flow cytometry. (F,G) H358 and A549 cells were incubated with the indicated concentrations of the Antp‐LP4 (●) or N‐Ter‐Antp (○) peptides in the appropriate serum‐free growth medium for 6 h, and cell death was analyzed by PI staining and flow cytometry. (H,I) PANC1 and noncancerous T‐Rex‐293 cells were incubated with the indicated concentrations of the Antp‐LP4 (●) or N‐Ter‐Antp (○) peptides in the appropriate serum‐free growth medium for 6 h, and cell death was analyzed by PI staining and flow cytometry.

**Figure 2:**
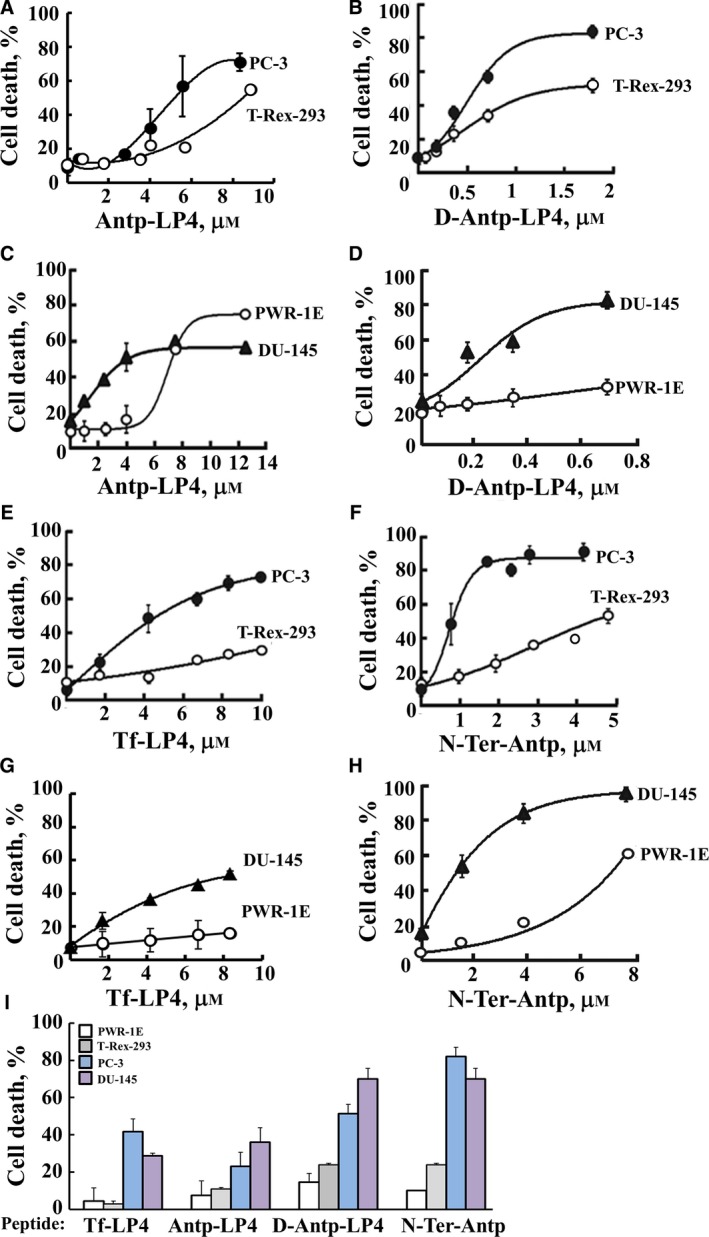
The VDAC1‐based peptides Antp‐LP4, D‐Antp‐LP4, Tf‐LP4, and N‐Ter‐Antp induce dramatic cell death in various prostate cancer cell lines but of noncancerous cells to a lesser extent. PC‐3 (●) and noncancerous T‐Rex‐293 cells (○) were incubated with the indicated concentrations of the Antp‐LP4 (A), D‐Antp‐LP4 (B), Tf‐LP4 (E), or N‐Ter‐Antp (F) peptides in the appropriate serum‐free growth medium for 6 h at 37 °C, and cell death was analyzed by PI staining and flow cytometry. DU‐145 cells (▲) and a noncancerous human prostate epithelial cell line, PWR‐1E (○), were incubated with the indicated concentrations of the Antp‐LP4 (C), D‐Antp‐LP4 (D), Tf‐LP4 (G), or N‐Ter‐Antp (H) peptides in the appropriate serum‐free growth medium for 6 h at 37 °C, and cell death was analyzed by PI staining and flow cytometry. (I) Representation of cell death induced with 4 μm of the indicated peptides (except for the D‐Antp‐LP4 peptide that was used at 0.7 μm due to its high potency) with PWR‐1E (white bars), T‐Rex‐293 (gray bars), PC‐3 (blue bars), and DU‐145 (purple bars) cells after accounting for the level of cell death in untreated cells. Data represent mean values ± SE (*n* = 3).

**Figure 3:**
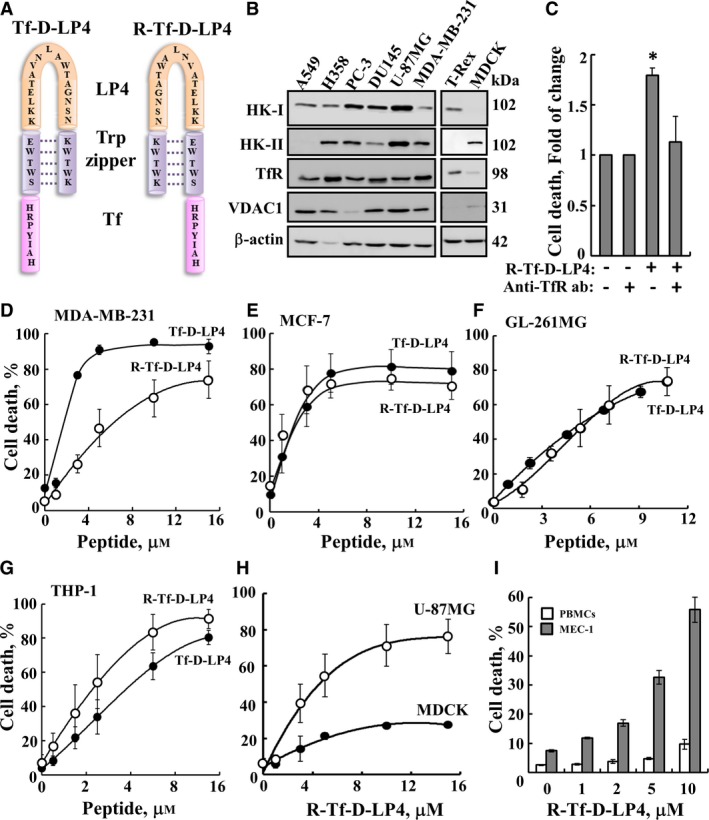
Cell death‐inducing activity of various VDAC1‐based peptides. (A) Schematic illustration of the structure of Tf‐D‐LP4 and R‐Tf‐D‐LP4 peptides. The VDAC1‐derived sequence LP4 is in orange, the cell‐penetrating sequence (Tf) is in pink, and the loop‐shaped stabilizing sequence, a tryptophan zipper (Trp zipper), is in purple. (B) Immunoblot analysis of HK‐I, HK‐II, TfR, and VDAC1 expression levels in representative cancer cell lines using specific antibodies. (C) R‐Tf‐D‐LP4 peptide entering the cells via the TfR receptor. U‐87MG cells were incubated with anti‐TfR antibodies (1 : 250) for 2 h followed by treatment with R‐Tf‐D‐LP4 peptide (5 μm) for an additional 3 h in the appropriate serum‐free growth medium, and cell death was analyzed by PI staining and flow cytometry. Representation of cell death induced with the peptide after accounting for the level of cell death in untreated cells. Data represent mean values ± standard deviation (*n* = 2) (*P ≤ 0.05). (D–F) Versions of the Tf‐LP4 peptide inducing cell death assayed using PI staining and flow cytometry. The R‐Tf‐D‐LP4 and Tf‐D‐LP4 peptides induce cell death of various cancer cell lines. MDA‐MB‐231 (D), MCF‐7 (E), GL‐261MG (F), and THP‐1 (G) cell lines were incubated for 5 h (90 min for THP‐1), with the indicated concentrations of the peptides R‐Tf‐D‐LP4 (○) or Tf‐D‐LP4 (●), and cell death was assayed. (H) R‐Tf‐D‐LP4 peptide induces cell death of U‐87MG cells but to a lesser extent of noncancerous MDCK cells. U‐87MG (○) and noncancerous MDCK (dog kidney epithelial) (●) cells were incubated for 5 h with the indicated concentrations of the R‐Tf‐D‐LP4 peptide, and cell death was assayed. Data represent mean values ± SE (*n* = 3). (I) R‐Tf‐D‐LP4 peptide specifically induces cancer cell death. PBMCs isolated from healthy individuals or MEC‐1 cells were incubated for 90 min with R‐Tf‐D‐LP4. Quantitation of FACS analysis of R‐Tf‐D‐LP4‐induced death of PBMCs (*n* = 3) and MEC‐1 cells (*n* = 4).

Forty‐one cancerous and five noncancerous cell lines were used in this study to examine the cell‐killing activity of the VDAC1‐based peptides Antp‐LP4, Tf‐LP4, Tf‐D‐LP4, R‐Tf‐D‐LP4, N‐Ter‐Antp, and others (Figs 1, 2, 3 and Tables 1, 2, 3). For each line, cells were incubated with different peptide concentrations, and cell death was determined by PI staining and FACS analysis (Figs 1, 2, 3). In addition, for each cell line, the concentration of the peptide inducing 50% cell death (EC_50_) was determined (Tables 1, 2, 3).

**Table 2:** Summary of the results with various peptides based on LP4 or the N‐terminal domain of VDAC1 inducing cell death. A549 or U‐87MG cells were incubated for 6 h with various concentrations (1–15 μm) of the peptide and analyzed for cell death by PI staining and FACS analysis. Values represent EC_50_ values (μm) (calculated from the maximal extent of cell death obtained). Results are the mean ± SE (*n* = 3)

No.	Peptide	Cell line	EC_50_, μm
1	Antp‐LP4	A549	6.4 ± 1.1
2	D‐Antp‐LP4	U‐87	6.5 ± 0.0
3	Tf‐D‐LP4	A549	1.3 ± 0.2
4	R‐Tf‐D‐LP4	A549	2.7 ± 0.5
5	N‐Ter‐Antp	A549 U‐87	10.2 ± 0.6 8.3 ± 1.5
6	Tf‐∆(1‐14)N‐Ter	U‐87	> 15
7	∆(1‐14)N‐Ter‐Tf	U‐87	> 15
8	N‐Ter∆21‐26‐Antp	U‐87	> 15
10	∆(1‐4)N‐Ter∆(21‐26)‐Antp	U‐87	> 15
11	∆(1‐9)N‐Ter∆(21‐26)‐Antp	U‐87	> 15
12	D‐∆(1‐16)N‐Ter‐Antp	U‐87	7.7
13	D‐∆1‐18N‐Ter‐Antp	U‐87	6.7 ± 1.4

**Table 3:** The Tf‐D‐LP4 and R‐Tf‐D‐LP4 peptides induce cell death in various cancer cell lines. Summary of the half‐maximal cell death activity (EC_50_) values (μm) (calculated from the maximal extent of cell death obtained) for cell death induction by the Tf‐D‐LP4 and R‐Tf‐D‐LP4 peptides in various cancer cell lines. Cell lines were incubated with different peptide concentrations for 90 min (for cells in suspension) or 6 h (for adherent cells). Cell death was determined by PI staining and FACS analysis. Data represent mean ± SE (*n* = 3–4). Mutation data taken from COSMIC (http://www.sanger.ac.uk/genetics/CGP/CellLines/). ND, not determined; NR, not reported

Cell line	Origin	Mutation status	Peptide EC_50_, μm	
			Tf‐D‐LP4	R‐Tf‐D‐LP4
U‐251MG	Human glioblastoma	TP53, PTEN, CDKN2A, NF1, PTPRT	1.3 ± 0.2	3.7 ± 0.7
U‐118MG	Human glioblastoma	TP53, PTEN, CDKN2A	1.6 ± 0.3	3.3 ± 0.9
U‐87MG	Human glioblastoma	PTEN, CDKN2A, KRAS, PCM1, NF1	1.5 ± 0.3	2.2 ± 0.4
LN‐18	Human glioblastoma	TP53, CDKN2A, JAK3, MLLT4, PTPRC	1.8 ± 0.4	3.3 ± 0.9
GL‐261MG	Mouse glioblastoma	TP53, KRAS	3.5 ± 0.3	3.6 ± 0.4
SH‐SY5Y	Human neuroblastoma	PTEN, CTNNA3, MCC, MTUS1	5.9 ± 1.2	> 15
N2A	Mouse neuroblastoma	NR	4.5 ± 0.5	9.4 ± 0.2
C6	Rat glioma	PTEN, CDKN2A, NK4A	10.0 ± 0.5	ND
PANC1	Human pancreas carcinoma	TP53, CDKN2A, KRAS	2.7 ± 0.9	3.5 ± 0.1
PANC2	Mouse pancreas	BRAF, SMAD4, NKX2‐1	ND	5.5 ± 0.7
SVR	Mouse pancreas	NR	ND	3.8
A‐375	Human melanoma	BRAF, MTOR, RUNX1, MECOM, MYH9	ND	2.0 ± 0.7
HTB‐66	Human melanoma	TP53, PTEN, BRAF, CDKN2A	ND	7.2
HTB‐72	Human melanoma	TP53, EGFR, BRAF	ND	5.7 ± 1.2
B16F10	Mouse melanoma	CDKN2A	3.7 ± 1.2	6.3 ± 1.3
B16F10.9	Mouse melanoma	CDKN2A	ND	5.2 ± 1.7
K1735 16	Mouse melanoma	NRAS, CDKN2A	ND	4.0 ± 0.6
K1735 M4	Mouse melanoma	NRAS, CDKN2A	4.6 ± 1.9	4.3 ± 0.3
MO1043	Human chronic lymphocytic leukemia	NR	3.5 ± 0.0	ND
CLL	Human chronic B‐cell leukemia	Patient‐specific mutations	1.6 ± 0.3	ND
MEC‐1	Human chronic B‐cell leukemia	TP53, PTEN, CDKN2A, BRCA1/2	1.5 ± 0.4	ND
THP‐1	Human acute monocytic leukemia	TP53, CDKN2A/B NRAS, MLLT4	5.8 ± 1.1	4.5 ± 1.5
MDA‐MB‐231	Human breast adenocarcinoma	TP53, KRAS, BRAF, CDKN2A, PDGFRA	2.0 ± 0.1	1.7 ± 0.8
MCF‐7	Human breast adenocarcinoma	CDKN2A, GATA3, BCR, FLT3, ERBB4	2.0 ± 0.6)	1.3 ± 0.3
TSA	Mouse breast cancer	ERBB2	3.2 ± 0.1	12.0 ± 3.5
A549	Human lung adenocarcinoma	KRAS, KEAP1, FLT3, ATR, CBL, STK11	1.3 ± 0.2	2.7 ± 0.5
HeLa	Human cervix adenocarcinoma	AR, TPR	2.5 ± 0.5	9.8 ± 1.4
PBC	Primary brain cell	NR	2.0 ± 0.0	6.3 ± 0.2
MDCK	Dog kidney epithelial	NR	ND	> 15

The results show that the cell death‐inducing activity of the peptides was cell type‐dependent. Accordingly, we subgrouped the cell lines into three groups according to their sensitivity to N‐Ter‐Antp (Fig. 1A) as highly sensitive (Group 1: EC_50_ = 0.9‐4.5 μm), sensitive (Group 2: EC_50_ of 6.5–10 μm), and less sensitive (Group 3: EC_50 _> 18 μm) (Table 1). Representative experiments assessing the cell death induction activity of the Antp‐LP4 and N‐Ter‐Antp peptides are shown for the B‐cell chronic lymphocytic leukemia (MEC‐1) and histiocytic leukemia (U‐937) cell lines, assigned to the highly sensitive group, where an EC_50_ ≤ 3 μm was measured (Fig. 1D,E). Melanoma (A375) and lung cancer cell lines (A549 and H358), representative of the sensitive group, presented an EC_50_ ≤ 6 μm (Fig. 1B,C,F,G), while the PANC1 pancreatic cancer cell line, representative of the much less sensitive group, presented an EC_50 _≥ 18 μm (Fig. 1H). Transformed noncancerous T‐Rex‐293 cells were assigned to the less sensitive group, and showed EC_50_ values of 18 and 29 μm for N‐Ter‐Antp and Antp‐LP4, respectively (Fig. 1I and Table 1). Thus, for both the Antp‐LP4 and N‐Ter‐Antp peptides, most of the cancer cell lines exhibited concentration‐dependent cell death following a 6‐h incubation, reaching a maximal level of cell death of over 90%. However, for the less sensitive group (Group 3), cell death was increased from about 10% in the absence of peptides to a steady‐state level of about 50% in their presence (Fig. 1H,I).

### 3.2 Evaluation of cell death induction by modified VDAC1‐based peptides

To increase VDAC1‐based peptide stability and specific targeting to cancer cells, we designed new versions of the LP4‐based peptide (Figs 2 and 3, and S1 and Tables 2 and 3). To protect the peptides from proteolytic degradation, non‐native D‐form amino acids were used to create the D‐Antp‐LP4 and D‐N‐Ter‐Antp peptides. The selectivity of these peptides for cancer cells was addressed by employing the transferrin receptor (hTfR)‐targeting peptide HAIYPRH (Tf) as a cell‐penetrating sequence. Tf was identified by phage display and shown to internalize conjugated compounds into the cytoplasm via the human hTfR (Oh *et al*., 2009), overexpressed in many cancers (Daniels *et al*., 2012) (Fig. 3B). Other modifications included a shortening of the VDAC1‐derived sequences from the C’ or N’ terminus (Table 2).

The ability of the N‐Ter‐Antp, Antp‐LP4, D‐Antp‐LP4, and Tf‐LP4 peptides to induce cell death and discriminate between cancer and noncancer cells was studied with the PC‐3 and DU‐145 prostate cancer cell lines and the noncancerous prostate epithelium PWR‐1E and T‐Rex‐293 cell lines (Fig. 2). The four tested peptides exhibited concentration‐dependent cell death induction in the prostate cancer cell lines. Sensitivity to the peptides was remarkably reduced in the noncancerous PWR‐1E and T‐Rex‐293 cell lines (Fig. 2). D‐Antp‐LP4 peptide was about fourfold more effective in inducing cell death of PC‐3 and DU‐145 cells than was the same peptide composed of L‐amino acids (compare Fig. 2A,C to B,D). This may have been due to increased protease protection of the former version of the peptide.

Tf‐LP4 was as active in inducing cell death as Antp‐LP4, yet was less effective than the N‐Ter‐Antp peptide (Fig. 2A,C,E–H). However, Tf‐LP4 showed almost no cell death induction in the noncancerous PWR‐1E and T‐Rex‐293 cell lines (Fig. 2E,G), pointing to its selectivity toward cancer cells. Similarly, N‐Ter‐Antp induced cell death in both the PC‐3 and DU‐145 cells but only to lesser extent in the noncancerous cell lines (Fig. 2F,H). The specificities of the various peptides toward prostate cancer cells are summarized in Fig. 2I.

Other versions of the N‐Ter‐Antp, Antp‐LP4 (Fig. 1A), Tf‐DLP4, and retro‐(R)‐Tf‐D‐LP4 (Fig. 3A) peptides were designed and tested with various cell lines (Fig. 3 and Tables 2 and 3) showing high expression levels of HK‐I, HK‐II, VDAC1, and TfR (Fig. 3B). These included short versions of N‐Ter‐Antp, such as D‐Δ(1‐14)N‐Ter‐Antp, D‐Δ(1‐16)N‐Ter‐Antp, D–Δ(1‐18)N‐Ter‐Antp, and Δ(21‐26)N‐Ter‐Antp, a peptide lacking the GXXXG motif at the C terminus (Fig. S1). The results clearly indicated that while the D–Δ(1–18)N‐Ter‐Antp peptide possesses similar (if not better) activity as the N‐Ter‐Antp peptide, deleting amino acids 21–26 in the GXXXG motif resulted in a nonactive peptide.

To address changes in orientation due to the D‐configuration of the amino acids, we also designed a retro‐inverso peptide. Retro‐inverso peptides are peptides in which the D‐amino acid sequence of the peptide is reversed, such that the α‐center chirality of the amino acids is also inverted. The reverse sequence helps maintain side‐chain topology similar to that of the original L‐amino acid peptide (Chorev, 2005; Holm *et al*., 2011; Liu *et al*., 2016).

To demonstrate the involvement of TfR in peptide cell penetration, we preincubated the cells with anti‐TfR antibodies prior to cells incubation with the R‐Tf‐D‐LP4 peptide (Fig. 3C). The results showed an about twofold decrease in Tf‐D‐LP4‐induced cell death in anti‐TfR antibody‐treated cells (Fig. 3C), suggesting the requirement of TfR for Tf‐D‐LP4‐mediated cell death.

Cell death induced by Tf‐D‐LP4 and R‐Tf‐D‐LP4 was tested in several cell lines. In MDA‐MB‐231 and MCF‐7 human breast cancer cells, R‐Tf‐D‐LP4 was less effective than Tf‐D‐LP4 (Fig. 3D,E), and in GL‐261MG mouse glioblastoma, they were similarly effective (Fig. 3F). In THP‐1 human acute monocytic leukemia cells, R‐Tf‐D‐LP4 was more effective than Tf‐D‐LP4 (Fig. 3G). The differences between the two versions of the peptide with respect to solubility and stability (Fig. S3) and, therefore, in their use *in vivo* will be described.

The results showed that these peptides induced cell death in all cancer cell lines tested (Figs 3D–G and S1), and to a lesser extent in noncancerous MDCK dog kidney epithelial cells (Fig. 3H) and in peripheral blood mononuclear cells (PBMCs) obtained from healthy individuals (Fig. 3I).

### 3.3 Characterization of the mode of action of VDAC1‐based peptides leading to cell death

**Figure 4:**
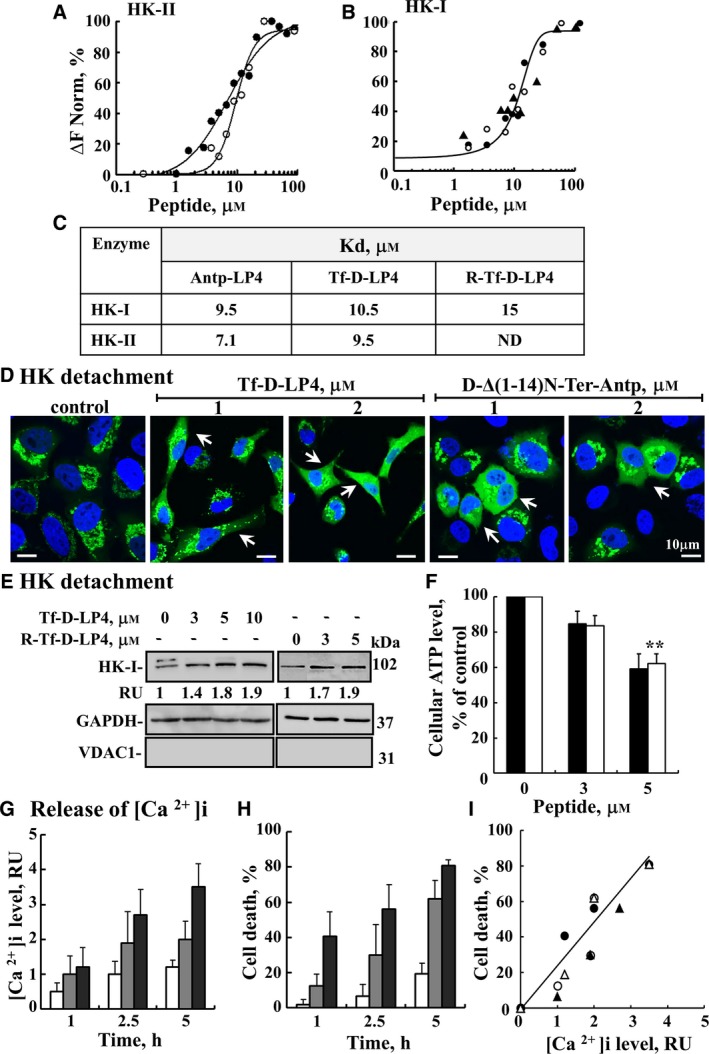
VDAC1‐based peptides act by interacting with and detaching HK, decreasing ATP levels, and releasing intracellular Ca^2+^. (A–C) The Antp‐LP4, Tf‐D‐LP4, and R‐Tf‐D‐LP4 peptides interact with HK‐I and HK‐II. Purified HK‐I and HK‐II were fluorescently labeled using the NanoTemper BLUE protein‐labeling kit. HK‐II (A) or HK‐I (B) (100 and 830 nm, respectively) was incubated with the Antp‐LP4 (●), Tf‐D‐LP4 (○), or R‐Tf‐D‐LP4 (▲) peptides (1–100 μm for 20 min). Then, 3–5 μL of the samples was loaded into MST‐grade glass capillaries, and thermophoresis was measured using a Monolith‐NT115 apparatus, as described in Materials and Methods. (C) Kd values of HK‐I and HK‐II for the different peptides. (D,E) The Tf‐D‐LP4, R‐Tf‐D‐LP4, and D‐∆(1‐14)N‐Ter‐Antp peptides induce HK detachment. (D) HeLa cells were transfected with plasmid pEGFP‐HK‐I and after 24 h, incubated with the indicated concentrations of the Tf‐D‐LP4 or D‐∆(1‐14)N‐Ter‐Antp peptides for 3 h in serum‐free medium. The final DMSO concentration in control and peptide‐treated cells was 0.07%. Fixed cells were visualized by confocal microscopy (Olympus 1X81). Arrows indicate cells with diffusion of HK‐I‐GFP. (E) A549 or HeLa cells were incubated with or without the indicated concentrations of the Tf‐D‐LP4 or R‐Tf‐D‐LP4 peptides, respectively, for 3 h, treated with digitonin (0.025%) and HK in the cytosolic fraction was analyzed by immunoblotting as described in Materials and Methods. Anti‐GAPDH and anti‐VDAC1 antibodies were used to verify the cytosolic and mitochondria extracts, respectively. The levels of HK‐I in the cytosolic fraction were quantified and are presented as relative units. (F) The R‐Tf‐D‐LP4 (black bars) and Tf‐D‐LP4 (white bars) peptides reduce cellular ATP levels assayed as described in Materials and Methods. Cellular ATP levels are presented as percentage of control. Results = mean ± SE (*n* = 3) **P ≤ 0.01. (G–I) The R‐Tf‐D‐LP4 peptide induces release of intracellular Ca^2+^. A549 cells were incubated with 3 μm (white bars), 5 μm (gray bars), or 10 μm (black bars) of the R‐Tf‐D‐LP4 peptide for 1, 2.5, or 5 h and harvested, and intracellular Ca^2+^ levels ([Ca^2+^]i) (G) and cell death (H) were assessed. [Ca^2+^]i levels and cell death were determined using Fluo‐4 and PI, respectively, and flow cytometry. (I) Correlation between cell death induced by 3 μm of the R‐Tf‐D‐LP4 (○) or 5 μm of the R‐Tf‐D‐LP4 (●) peptides and the decrease in [Ca^2+^]i levels induced by a 2.5‐h incubation with R‐Tf‐D‐LP4 (▲) or a 5‐h incubation with R‐Tf‐D‐LP4 (▵).

**Figure 5:**
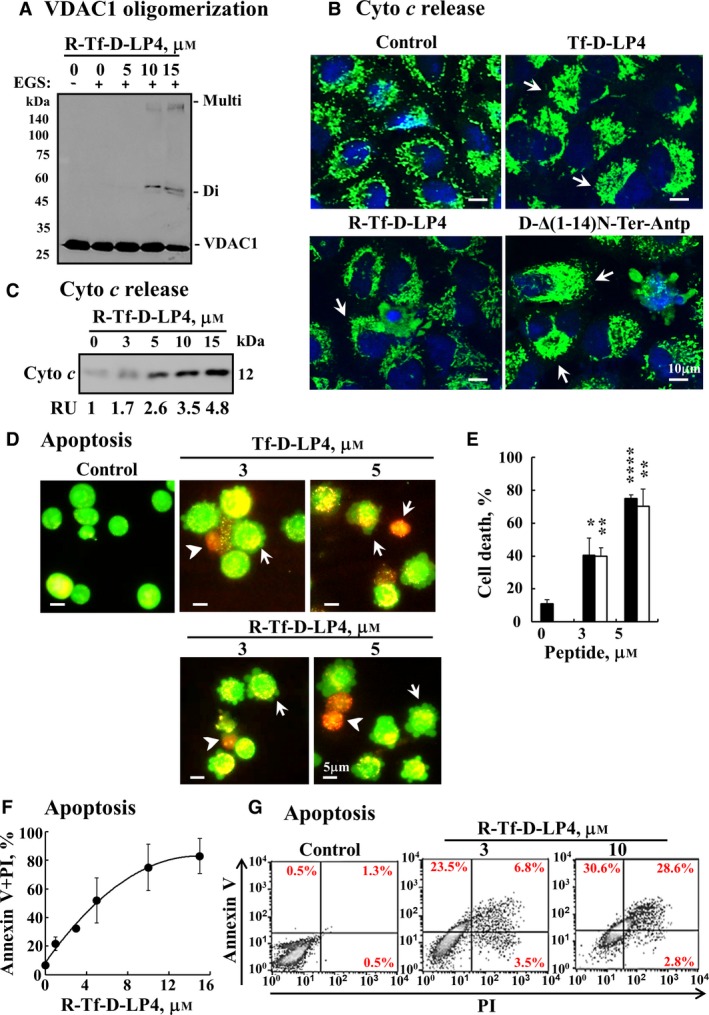
VDAC1‐based peptides act by inducing VDAC1 oligomerization, releasing Cyto *c*, and inducing apoptosis. (A) R‐Tf‐D‐LP4 induces VDAC1 oligomerization. HeLa cells were incubated with the indicated concentrations of R‐Tf‐D‐LP4 for 5 h, followed by incubation with EGS (300 μm, 15 min) and analysis of VDAC1 oligomerization. ‘Di’ indicates dimeric VDAC1, and ‘Multi’ indicates high oligomeric states of VDAC1. (B,C) The Tf‐D‐LP4, R‐Tf‐D‐LP4, and D‐∆(1‐14)N‐Ter‐Antp peptides induce Cyto *c* release. (B) A549 cells were incubated with the Tf‐D‐LP4, R‐Tf‐D‐LP4, or D‐∆(1‐14)N‐Ter‐Antp peptides (10 μm) for 6 h in serum‐free medium. Release of Cyto *c* from the mitochondria was analyzed by immunostaining using anti‐Cyto *c* antibodies and confocal microscopy (Olympus 1X81). Arrows indicate cells showing diffusion of Cyto *c*. (C) HeLa cells were incubated with or without the indicated concentration of the R‐Tf‐D‐LP4 peptide, respectively, for 3 h, treated with digitonin (0.025%), and Cyto *c* in the cytosolic fraction was analyzed by immunoblotting as described in Materials and Methods. Anti‐GAPDH and anti‐VDAC1 antibodies were used to identify the cytosolic and mitochondria extracts, respectively (not shown). The levels of Cyto *c* in the cytosolic fraction were quantified and are presented as relative units. (D–E) The Tf‐D‐LP4 and R‐Tf‐D‐LP4 peptides induce apoptotic cell death. A549 cells were treated with the indicated concentrations of the Tf‐D‐LP4 or R‐Tf‐D‐LP4 peptides for 3 h and then stained with acridine orange and ethidium bromide (100 μg·mL^−1^). Arrows and arrowheads indicate cells with membrane blebbing (early apoptotic state) and cells in a late apoptotic state, respectively. (E) Quantitation of apoptosis in experiment D with Tf‐D‐LP4 or R‐Tf‐D‐LP4‐treated cells as black and white bars, respectively. Results = mean ± SE (*n* = 3) (*P ≤ 0.05, **P ≤ 0.01, ****P ≤ 0.0001). (F) The R‐Tf‐D‐LP4 peptide induces apoptotic cell death. A549 cells were incubated for 3 h with the indicated concentrations of the R‐Tf‐D‐LP4 peptide in serum‐free growth medium at 37 °C with DMSO at a final concentration of 0.15% in both control and peptide‐treated cells. Cells were subjected to FITC–annexin V/PI staining and flow cytometry analysis. (G) Representative FACS analysis of DMSO (control)‐ or R‐Tf‐D‐LP4 (3, 10 μm)‐treated A549 cells.

The mode of action of the VDAC1‐based peptides from the second stage of optimization was examined as was that of the first versions of the peptides (Abu‐Hamad *et al*., 2008; Arzoine *et al*., 2009). This included assessing detachment of mitochondria‐bound HK, changes in cellular ATP and Ca^2+^ levels, and induction of Cyto *c* release and apoptosis (Figs 4 and 5).

Recombinant, purified HK‐I and HK‐II directly interacted with the Antp‐LP4, Tf‐D‐LP4, and R‐Tf‐D‐LP4 peptides with similar binding affinities (Fig. 4A–C). Next, displacement of mitochondrial‐bound HK induced by the Tf‐D‐LP4 and D‐Δ(1‐14)N‐Ter‐Antp peptides was demonstrated by immunofluorescence. These efforts showed that in untreated cells, HK‐I‐GFP fluorescence was punctuated, indicating mitochondrial HK‐I‐GFP distribution, while in cells incubated with the Tf‐D‐LP4 or D‐Δ(1‐14)N‐Ter‐Antp peptides, the fluorescence was diffuse, reflecting displacement of HK from the mitochondria and its presence in the cytosol (Fig. 4D). HK detachment induced by the Tf‐D‐LP4 and R‐Tf‐D‐LP4 peptides was also demonstrated by cell fractionation and immunoblotting, showing an about twofold increase in HK in the cytosolic fraction derived from peptide‐treated cells (Fig. 4E).

The effects of the Tf‐D‐LP4 and R‐Tf‐D‐LP4 peptides on cellular ATP and intracellular Ca^2+^ ([Ca^2+^]i) levels were also analyzed (Fig. 4F–I). In peptide‐treated cells, cellular ATP levels were decreased by about 40% (Fig. 4F), suggesting that mitochondrial energy production was impaired by the peptides. As apoptosis induction by various stimuli was shown to disrupt cellular Ca^2+^ homeostasis (Florea and Busselberg, 2009; Keinan *et al*., 2013; Weisthal *et al*., 2014), the effect of the R‐Tf‐D‐LP4 peptide on [Ca^2+^]i was tested using Fluo‐4 (Fig. 4G,I). R‐Tf‐D‐LP4 increased [Ca^2+^]i in a concentration‐ and time‐dependent manner (Fig. 4G,I). Moreover, a linear correlation between the increase in [Ca^2+^]i (Fig. 4G) and cell death (Fig. 4H) was obtained (Fig. 4I).

Next, we analyzed the effects of the VDAC1‐based peptides on several apoptosis‐associated features, such as VDAC1 oligomerization, Cyto *c* release, and apoptotic cell death (Fig. 5). Apoptosis induction by various apoptosis inducers has been shown to lead to VDAC1 oligomerization, regardless of the cell type or apoptosis inducer used (Keinan *et al*., 2010, 2013). Similarly, cell treatment with the peptides led to VDAC1 oligomerization (Fig. 5A). Peptide‐induced Cyto *c* release was demonstrated by immunofluorescence using anti‐Cyto *c* antibodies and confocal microscopy (Fig. 5B). Representative images of control cells show punctuated fluorescence, suggesting mitochondrial distribution of Cyto *c,* and while in peptide‐treated cells, the fluorescence was more diffuse, pointing to the release of Cyto *c* (Fig. 5B). Cell fractionation and immunoblotting analysis demonstrated Cyto *c* to be in the cytosolic fraction of only peptide‐treated cells (Fig. 5C).

Finally, apoptotic cell death was analyzed using acridine orange/ethidium bromide staining, as well as annexin V–FITC and PI staining and FACS analysis (Figs 5D–G and S2). Cell incubation with the Tf‐D‐LP4 or R‐Tf‐D‐LP4 peptides induced membrane blebbing, chromatin condensation, and orange staining of nuclei, reflecting a late apoptotic stage (Figs 5D and S2A). Quantitation of these results showed that the peptides induced over 70% apoptotic cell death in A549 cells (Fig. 5E). Apoptosis was also revealed by annexin V–FITC and PI staining, with representative FACS results and their quantitation showing that the R‐Tf‐D‐LP4 peptide induced over 90% apoptotic cell death (Figs 5F,G and S2B,C).

These results indicate that the peptides induced VDAC1 oligomerization, Cyto *c* release, and subsequently apoptosis, as shown previously for other versions of VDAC1‐based peptides in studies of CLL and GBM (Prezma *et al*., 2013; Shteinfer‐Kuzmine *et al*., 2017).

Due to the advantageous properties of R‐Tf‐D‐LP4 relative to Tf‐D‐LP4, including inhibition of purified HK‐I activity (Fig. S3A,B), increased solubility (Fig. S3C), and more stability with respect to temperature and incubation time (Fig. S3D,E), the former was selected for *in vivo* experiments.

### 3.4 VDAC1‐based peptides inhibit *in vivo* growth of glioblastoma, lung, and breast cancer tumors

**Figure 6:**
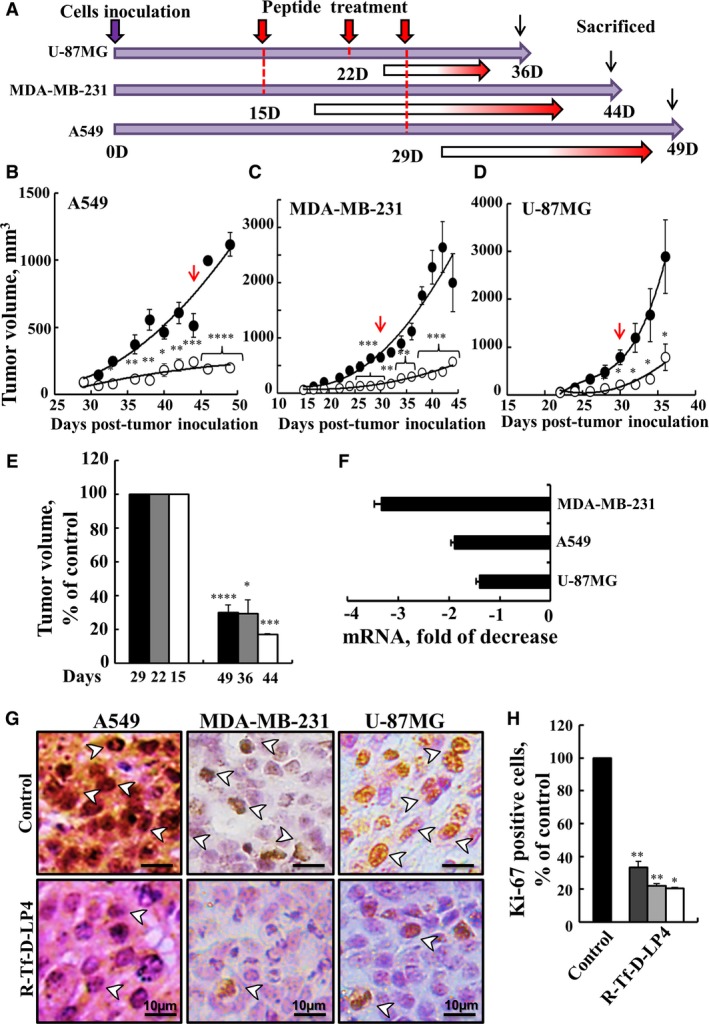
The R‐Tf‐D‐LP4 peptide inhibits tumor growth and cell proliferation in lung and breast cancers and in a GBM xenograft mouse models. (A) Graphical representation of the xenograft experiment protocol used. (B–D) The R‐Tf‐D‐LP4 peptide inhibits tumor growth. A549 (B), MDA‐MB‐231 (C), or U‐87MG (D) cells were inoculated into male nude mice. When tumor volume was 50–100 mm^3^ (on day 15 for MDA‐MB‐231, day 22 for U‐87MG, and day 29 for A549 cells), the mice were subdivided into two matched groups (five mice per group) and injected every two days with HBSS (●, control), or with the R‐Tf‐D‐LP4 peptide (○, 40 or 60 μm, indicated by red arrow). The final DMSO concentration in both tumor groups was 0.05%. The calculated average tumor volumes are presented as means ± SE (*n* = 5) (*P ≤ 0.05, **P ≤ 0.01; ***P ≤ 0.001; ****P ≤ 0.0001). (E) The volume of the R‐Tf‐D‐LP4‐TTs in A549 (black bars), U‐87MG (gray bars), and MDA‐MB‐231 (white bars) is presented as % of control for the indicated selected time. Results = mean ± SE (*n* = 5) (*P ≤ 0.05, ***P ≤ 0.001, ****P ≤ 0.0001). (F–H) The R‐Tf‐D‐LP4 peptide decreases cell proliferation. (F) RNA extracts obtained from control tumors and R‐Tf‐D‐LP4‐TTs of A549, MDA‐MB‐231, or U‐87MG cells were analyzed by qPCR using specific primers for Ki‐67 (*n* = 3). (G) Representative sections from control tumors and R‐Tf‐D‐LP4‐TTs were immunostained for Ki‐67, hematoxylin‐stained, and visualized by light microscopy. Arrowheads indicate representative cells with nuclear localization of Ki‐67. (H) Quantitative analysis of Ki‐67‐positive cells from control tumors and R‐Tf‐D‐LP4 peptide‐TTs of A549 (dark gray bars), U‐87MG (light gray bars), or MDA‐MB‐231 (white bars) cells. Results = mean ± SE (*n* = 3) (*P ≤ 0.05 or **P ≤ 0.01).

Next, we tested the effects of the R‐Tf‐D‐LP4 peptide *in vivo,* as it was more soluble than the Tf‐D‐LP4 peptide and inhibited HK activity (Fig. S3). The effects of the R‐Tf‐D‐LP4 peptide on glioblastoma‐derived U‐87MG, lung cancer‐derived A549, or breast cancer‐derived MDA‐MB‐231 cell xenograft mouse models were tested according to an established protocol (Fig. 6A). Nude mice were s.c. injected with the cells, and following tumor formation to a volume of 50–100 mm^3^, the mice were split into two tumor volume‐matched groups. The control group was treated intratumorally with HBSS, while the second group was treated with the R‐Tf‐D‐LP4 peptide (40 or 60 μm), with the calculated DMSO final concentration in both groups being 0.05%, and tumor growth was followed (Fig. 6B–D). In the three cancer models, untreated tumor volumes grew exponentially and increased over the course of the experiment, while the volume of peptide‐treated tumors (TTs) only slightly increased. A decrease of 70–80% in tumor volume was obtained in the three tumor types upon peptide treatment (Fig. 6E).

After sacrificing the mice, tumors were excised and each half tumor was fixed with formaldehyde and used for hematoxylin–eosin (H&E) and IHC staining, while the second half was frozen and used for RNA isolation and qPCR. Control tumors and R‐Tf‐D‐LP4‐TTs were analyzed by qPCR using specific primers for cell proliferation factor Ki‐67. The results showed a decrease of two‐ to threefold in Ki‐67 mRNA levels in peptide‐TTs (Fig. 6F). Ki‐67 levels were also analyzed from paraffin‐embedded sections, showing strong staining of nuclei in the three types of untreated tumors, while staining in the R‐Tf‐D‐LP4‐TTs was pronouncedly low (Fig. 6G). Quantitative analysis indicated that Ki‐67‐positive cell numbers were lower by 70‐80% in the R‐Tf‐D‐LP4‐TTs, relative to the control tumors (Fig. 6H).

**Figure 7:**
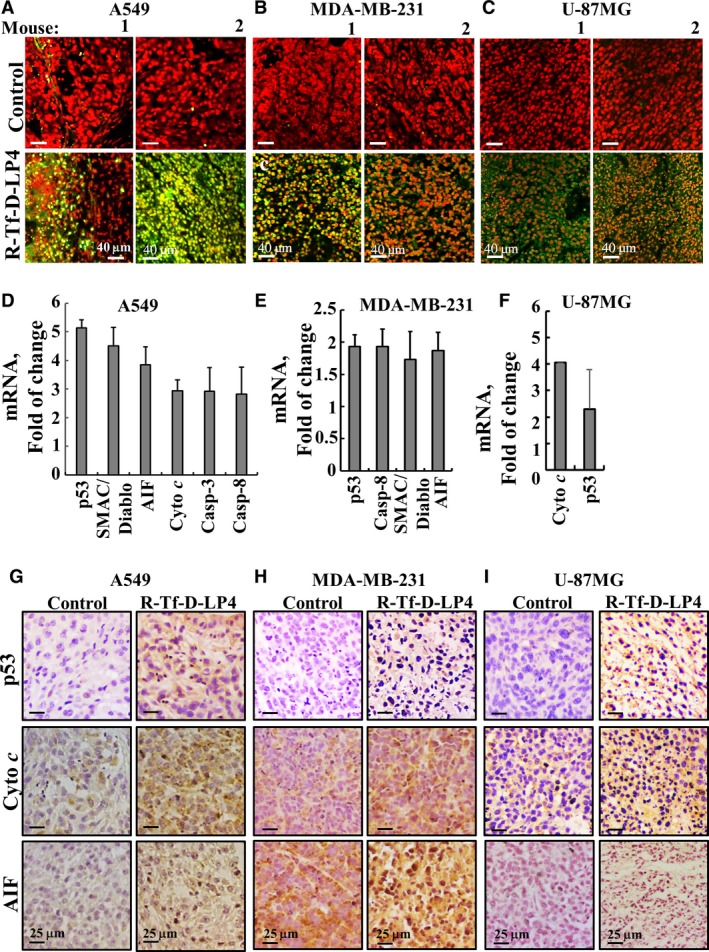
Tumor treatment with the R‐Tf‐D‐LP4 peptide induced apoptosis and enhanced the expression of apoptosis‐related proteins in lung and breast cancers and GBM xenograft mouse models. (A–C) TUNEL staining on paraffin‐embedded sections cut from control tumors and R‐Tf‐D‐LP4‐TTs, each derived from tumors dissected from two mice. A549 (A), MDA‐MB‐231 (B), or U‐87MG (C) xenografts. Red indicates PI nuclear staining and green indicates TUNEL staining. (D–F) RNA extracts obtained from control tumors and R‐Tf‐D‐LP4‐TTs from A549 (D), MDA‐MB‐231 (E), or U‐87MG (F) were analyzed by qPCR for p53, SMAC/Diablo, AIF, Cyto *c*, caspase‐3, or caspase‐8 (*n* = 3). (G–I) Representative sections from control and R‐Tf‐D‐LP4‐treated tumors from A549 (G), MDA‐MB‐231 (H), or U‐87MG (I) cells were immunostained for p53, Cyto *c* or AIF, hematoxylin‐stained and visualized by light microscopy.

As the mode of action of the peptide involves apoptosis induction, we analyzed the presence of apoptotic cells in the R‐Tf‐D‐LP4‐TTs *in situ* using TUNEL staining (Fig. 7A–C). While no TUNEL‐positive cells were apparent in control HBSS‐TTs, the majority of the cells were TUNEL‐positive in the R‐Tf‐D‐LP4‐TTs, with staining colocalizing with PI nuclear staining. The results clearly indicate that the peptide induced apoptotic cell death. Thus, the marked decrease in tumor size in the peptide‐treated xenografts can be attributed both to inhibition of cell proliferation (Fig. 6F–H) and to peptide‐induced cell death (Fig. 7A–C).

Furthermore, in A549‐, MDA‐MB‐231‐, and U‐87MG‐derived tumors treated with the R‐Tf‐D‐LP4 peptide, p53 expression levels were highly elevated (over two‐ to fivefold) (Fig. 7D–F). Caspase‐8, mediating the extrinsic apoptosis pathway, and caspase‐3 were also overexpressed (two‐ to threefold) in R‐Tf‐D‐LP4‐TTs (Fig. 7D,E). Cyto *c*, a key player in mitochondria‐mediated apoptosis, was also highly expressed (two‐ to fourfold) in the R‐Tf‐D‐LP4‐TTs (Fig. 7D,F). Similarly, expression levels of the pro‐apoptotic protein SMAC/Diablo were much higher (two‐ to fivefold) in R‐Tf‐D‐LP4‐TTs (Fig. 7D,E). Similar results were obtained with other VDAC1‐based peptides, with Δ(1‐14)N‐Ter‐Antp‐TTs and Tf‐D‐LP4‐TTs showing elevated levels of apoptosis‐related proteins, such as Cyto *c*, p53, and caspases (Shteinfer‐Kuzmine *et al*., 2017),

These results were further confirmed when tumor sections were IHC‐stained with specific antibodies against the pro‐apoptotic proteins p53, Cyto *c*, and AIF. In all cases, a massive increase in staining intensity was seen in R‐Tf‐D‐LP4‐TTs (Fig. 7G–I). The overexpression of pro‐apoptotic proteins may explain the high level of apoptosis obtained in the R‐Tf‐D‐LP4‐TTs.

### 3.5 R‐Tf‐D‐LP4 peptides alter the expression levels of metabolic enzymes and transporters *in vivo*

**Figure 8:**
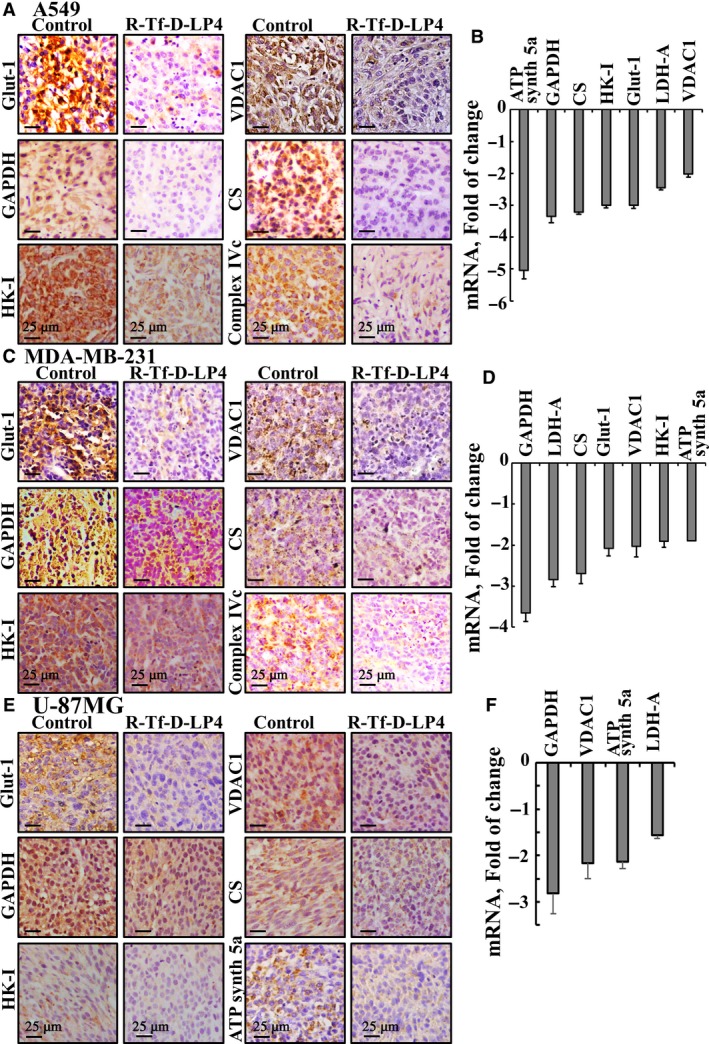
R‐Tf‐D‐LP4‐TTs show decreased expression of energy‐ and metabolism‐related enzymes in lung and breast cancers and GBM xenograft mouse models. (A,C,E) Representative sections from control tumors and R‐Tf‐D‐LP4‐TTs derived from A549 (A), MDA‐MB‐231 (C), or U‐87MG (E) cells were IHC‐stained for Glut‐1, GAPDH, HK‐I, VDAC1, CS, complex IVc, and ATP synthase 5a. Sections were also hematoxylin‐stained and visualized by microscopy. (B,D,F) RNA was isolated from control tumors and R‐Tf‐D‐LP4‐TTs derived from A549 (B), MDA‐MB‐231 (D), or U‐87MG (F) cells and analyzed by qPCR for the expression of ATP synthase 5a, GAPDH, CS, HK‐I, Glut‐1, LDH‐A, and VDAC1.

Next, we analyzed the effect of R‐Tf‐D‐LP4 treatment on the metabolic features of residual tumors derived from A549, MDA‐MB‐231, and U‐87MG cells using IHC and qPCR (Fig. 8). The R‐Tf‐D‐LP4 peptide decreased the expression of metabolism‐related proteins in A549‐, MDA‐MB‐231‐, and U‐87MG‐derived tumors, as analyzed by IHC and qPCR (Fig. 8). In the R‐Tf‐D‐LP4‐TTs, the expression levels of the glucose transporter (Glut‐1) and of the glycolytic enzymes HK‐I and glyceraldehyde dehydrogenase (GAPDH) were dramatically decreased, with qPCR analysis showing decreases of three‐ to fourfold. The expression levels of mitochondrial proteins associated with metabolism were also decreased. The level of VDAC1, a key protein in cancer cell energy and metabolism homeostasis, was also decreased, as were the levels of the Krebs cycle enzyme citrate synthase (CS), the electron transport chain protein complex IV, and ATP synthase subunit 5a (Fig. 8).

As cancer cells undergo metabolic reprograming essential for cancer cell survival and growth (Vander Heiden *et al*., 2009), these findings point to a reversal of the metabolic reprograming of the cancer cells, originated from different cancer types, in response to R‐Tf‐D‐LP4 peptide action.

### 3.6 The R‐Tf‐D‐LP4 peptide decreased the expression levels of stem cells markers

**Figure 9:**
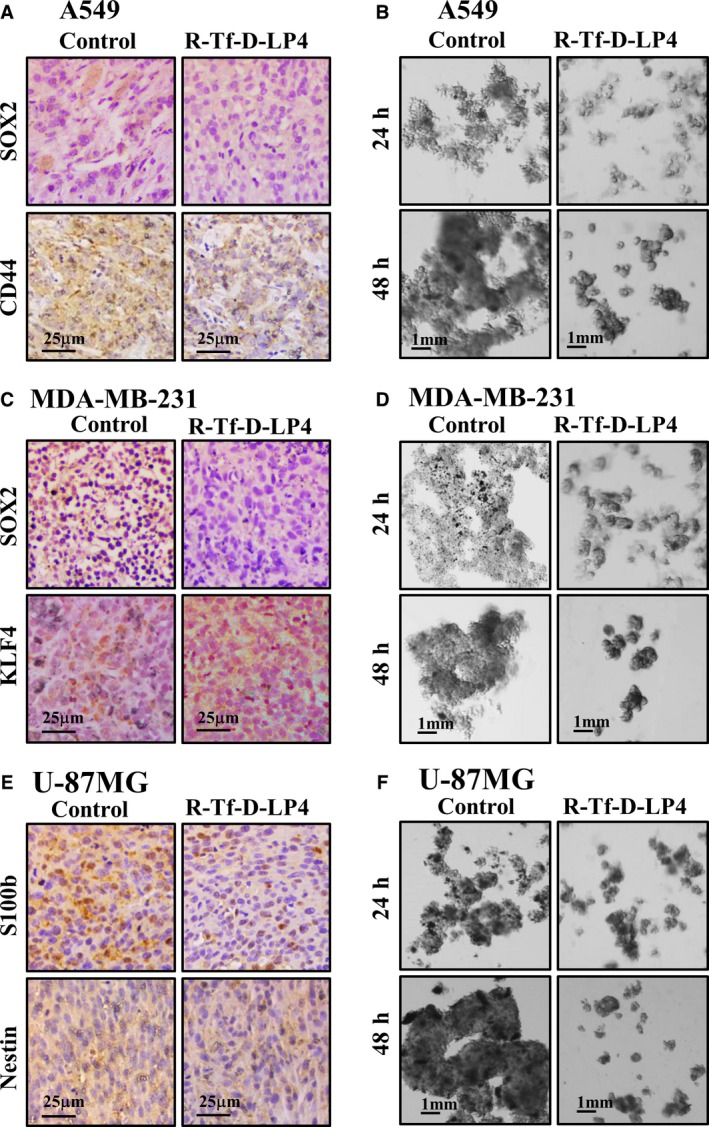
R‐Tf‐D‐LP4‐TTs show decreased expression of cancer stem cell markers in lung and breast cancers and GBM xenograft mouse models and spheroid formation. (A,C,E) IHC staining of paraffin‐embedded sections derived from control tumors and R‐Tf‐D‐LP4‐TTs obtained from A549 (A), MDA‐MB‐231 (C), and U‐87MG (E) xenografts for SOX2, CD44, KLF4, S100b, and Nestin. The selected CSC markers for each cancer type are indicated. (B,D,F) For spheroid formation, A549 (B), MDA‐MB‐231 (D), and U‐87MG (F) cells were incubated with R‐Tf‐D‐LP4 (15 μm) for 24 and 48 h in stem cell growth medium and assayed as described in Materials and Methods and visualized by light microscopy.

Cancer stem cell (CSC) populations have been identified in nearly all human malignancies, including those in the brain, pancreas, ovary, colon, and liver, as well as leukemia (Wang *et al*., 2011). CSCs are involved in tumor growth and disease progression and are thought to be highly resistant to a number of chemotherapeutic and radiation therapeutic strategies (Bao *et al*., 2006). We have previously demonstrated that glioblastoma cancer stem cells were eliminated by Tf‐D‐LP4 tumor treatment. Thus, we asked whether the R‐Tf‐D‐LP4 peptide affects the expression of stem cells markers specific to each cancer type (Fig. 9).

For GBM, we confirmed our previous results (Shteinfer‐Kuzmine *et al*., 2017), with R‐Tf‐D‐LP4 peptide treatment eliminating the expression of S100b and Nestin (Fig. 9E). Lung cancer stem cell markers include the transcription factor SOX2, the metabolic marker aldehyde dehydrogenase isoform 1 (ALDH1), and the cell surface markers CD133, CD44, CD166, and ABCG2 (Ho *et al*., 2007; O'Flaherty *et al*., 2012). R‐Tf‐D‐LP4‐TTs showed decreased expression of the tested markers CD44 and SOX2 (Fig. 9A). Breast CSC‐specific markers include KLF4, BMI‐1, CD44, CD24, CD49f, ALDHA1, and EpCAM (La Porta and Zapperi, 2013). R‐Tf‐D‐LP4‐TTs showed decreased expression of KLF4 and SOX2 (Fig. 9C). Thus, R‐Tf‐D‐LP4 peptide eliminated stem cells in all cancer types tested.

As tumor spheroid formation (clonogenicity) is a well‐known cancer stem cell feature and is considered as the gold standard *in vitro* method for evaluating the presence of CSCs (Valent *et al*., 2012; Weiswald *et al*., 2015), we performed this assay using the three cell lines used in the *in vivo* study. Using stem cell‐specific medium (Pollard *et al*., 2009), we tested the effect of R‐Tf‐D‐LP4 cell treatment on spheroid/aggregate formation and found that the peptide attenuated their formation, in comparison with peptide‐untreated lung and breast cancer and GBM cell lines (Fig. 9B,D,F).

## 4 Discussion

Many malignant cells arise from the multistep process of tumorigenesis, involving accumulation of inherited or acquired genetic abnormalities, some of which drive malignant cells to reprogram energy production and afford protection from apoptosis, a hallmark of the majority of cancer types. Newly developed targeted therapies for cancer, including immunotherapy and anti‐angiogenesis strategies, have shown that 1/3 of tumors respond to immunotherapy, and even less to angiogenesis inhibition, with patients showing varied responses, relapse, and resistance (Arbiser *et al*., 2017). Given these findings, and considering the heterogeneity and complexity of malignant tumors, a pandrug that acts regardless of the origin of the cancer and the mutations involved and which targets major cancer hallmarks, such as reprogramed metabolism and apoptosis, is required.

VDAC1, as mitochondrial gatekeeper, functions in energy production, Ca^2+^ homeostasis, oxidative stress, and apoptosis and as a hub protein interacting with over 150 proteins, including anti‐apoptotic proteins (Shoshan‐Barmatz *et al*., 2010, 2017a,b). Hence, targeting the interactions of VDAC1 with anti‐apoptotic proteins (Bcl‐2, Bcl‐xL) or proteins supporting the metabolic requirements of cancer cells (HK‐I, HK‐II) is a promising strategy for the development of anticancer agents.

We have developed several VDAC1‐based peptides that act as a ‘decoy’ to trap VDAC1‐interacting proteins, thus competing with VDAC1 for interaction with Bcl‐2, Bcl‐xL, and HK and consequently interrupting their anti‐apoptotic activities (Arbel and Shoshan‐Barmatz, 2010; Arbel *et al*., 2012). The VDAC1‐based peptides are discussed below with respect to the mode of cell‐killing action, specificity to cancer cells, effectiveness in cancer mouse models, and advantages as potential anticancer drugs.

### 4.1 VDAC1‐based peptide optimization and their cancer cell death activation, regardless of mutational status

Cell‐penetrating VDAC1‐based N‐Ter‐Antp, Tf‐D‐LP4, and Antp‐LP4 peptides and their improved derivatives selectively promoted cancer cell death in over 40 genetically characterized cancer cell lines, derived from different human malignancies, and possessing different pathways for apoptosis evasion (Figs 1, 2, 3 and S1 and Tables 1, 2, 3). VDAC1‐based peptides induced cell death in both p53‐positive and p53‐defective cancer cell lines (e.g., U‐87MG and MCF‐7 or MDA‐MB‐231 and HTB‐66), and sensitivity to the peptides was independent of tumor origin (adenocarcinoma or carcinoma).

The results on cell death induced by the N‐Ter‐Antp and Antp‐LP4 peptides point to three levels of sensitivity, as reflected by EC_50_ values (Table 1). The highly sensitive group (EC_50_ < 0.9–4.5 μm, 90‐min incubation) includes hematological malignancies (e.g., myelogenous leukemia, histiocytic lymphoma, CLL, acute T‐cell leukemia) and the prostate carcinoma cell lines (PC‐3 and DU‐145). The second‐most sensitive group (EC_50_, 6.5–10_ _μm) includes A549 and H358 lung cancer cells. The least sensitive group, with EC_50_ values 20‐ to 30‐fold higher than for the highly sensitive group, includes the PANC1 pancreatic cancer cell line and noncancerous PWR‐1E and T‐Rex‐937 cells and PBCs and PBMCs. The different sensitivities of the various cancer cell types to the peptides could result from differences in anti‐apoptotic protein expression levels. For example, the most peptide‐sensitive cells are those expressing mutated p53 and very high levels of HK‐II (Tables 1 and 3).

The N‐Ter‐Antp, Antp‐LP4, and Tf‐LP4 peptides were further optimized (Figs 3 and S1, and Table 2) with respect to sequence length, D *vs*. L amino acid conformation, cell‐penetrating peptide (CPP) type, and C‐ or N‐terminal position. The N‐Ter peptide was active only when Antp was used as the CPP but not Tf, a sequence recognized by the TfR. Moreover, deletion of the GXXXG motif (amino acids 21–26), shown to be involved in protein–protein interaction (Cymer *et al*., 2012), resulted in a nonactive peptide. The N‐Ter‐Antp peptide was active even after shortening the N terminus of the peptide by up to 18 of the 26 amino acids comprising the peptide (Fig. S1C).

As expected for a protease‐protected peptide, D‐Antp‐LP4 showed the highest efficacy in killing prostate cancer cells, with an EC_50_ in the submicromolar range, 8‐ to 30‐fold less than that of the L‐amino acid‐based version (Fig. 2).

The better effectiveness of Tf‐LP4 and its retro‐version R‐Tf‐D‐LP4 in inducing cell death than Antp‐LP4 peptides (Table 2) may result from TfR being highly expressed in cancer cells (Daniels *et al*., 2012) (Fig. 3B). As R‐Tf‐D‐LP4 was more soluble and stable than Tf‐D‐LP4 (Fig. S3C–E), and inhibited HK activity (Fig. S3A, B), it was selected for *in vivo* studies.

The findings that the VDAC1‐based N‐Ter‐Antp, Antp‐LP4, Tf‐LP4, and, particularly, the protease‐protected D‐version of the peptides (Figs 1, 2, 3) efficiently killed such varied cancer cells, regardless of the cell mutation status, provide the opportunity for developing new therapies for different cancers.

### 4.2 Modes of action and advantages of VDAC1‐based peptides

Our results with cells in culture and in tumors suggest that the VDAC1‐based peptides have a triple mode of action, namely affecting cell energy production and metabolism, preventing the protective effect of anti‐apoptotic proteins, and inducing apoptosis (Figs 4, 5 and S2). Cancer cells possess reprogramed metabolism, with the high use of glucose as an energy source via glycolysis and involving elevated expression of genes encoding glucose transporters and glycolytic enzymes, as HK, being a feature common to most solid tumors (Hanahan and Weinberg, 2011; Kaelin and Thompson, 2010; Koppenol *et al*., 2011).

Here, we demonstrated that our peptides (Antp‐LP4, Tf‐D‐LP4, and R‐Tf‐D‐LP4) interacted with purified HK‐I and HK‐II (Fig. 4A–C), detaching bound HK‐I (Fig. 4D,E). Thus, these ‘decoy’ peptides impaired the HK‐VDAC1 interaction, leading to HK detachment and affecting the energy balance of high energy‐demanding cancer cells. By detaching HK that catalyzes the first step of glycolysis, glycolysis is inhibited. Consequently, the TCA cycle is slowed as less acetyl‐CoA is produced from pyruvate. As mitochondrial ATP is the preferential substrate for bound HK, detachment of mitochondria‐bound HK decreased cellular ATP levels (Fig. 4F); this precedes a decrease in cell viability (Machida *et al*., 2006). Moreover, as the interaction of VDAC1 with HK mediates coupling between OXPHOS and glycolysis, enhanced cholesterol synthesis, decreased ROS production, and protection against apoptosis, disrupting this interaction should have multiple effects on cancer cells (Shoshan‐Barmatz *et al*., 2010, 2017a,b). Accordingly, detachment of HK from VDAC1 represents a novel therapeutic strategy to impair cancer metabolism and augment apoptosis (Krasnov *et al*., 2013; Shoshan‐Barmatz *et al*., 2015).

A number of agents, including 2‐deoxyglucose (2‐DG), 3‐bromopyruvate (3‐BP), an alkylating reagent, and lonidamine, have been used to inhibit HK activity and disrupt glycolysis (Krasnov *et al*., 2013). In addition, the antifungal agents clotrimazole and bifonazole were shown to disrupt the HK‐VDAC1 complex (Goldin *et al*., 2008; Penso and Beitner, 1998). However, these agents are not very potent, not targeted to cancer cells, and they are toxic to certain normal tissues where glucose is the main energy source, such as brain, retinae, and testes (Krasnov *et al*., 2013). In contrast, the Tf‐D‐LP4 and R‐Tf‐D‐LP4 peptides are targeted to cancer cells overexpressing TfR and thus specifically disrupt the HK‐VDAC1 interaction in cancer cells and affect overall cellular bioenergetics, as well as activating apoptosis (Figs 4, 5 and S2).

The R‐Tf‐D‐LP4 but not the Tf‐D‐LP4 peptide inhibits HK activity (Fig. S3). As HK is a cellular protein, its inhibition by the R‐Tf‐D‐LP4 peptide takes place in cells overexpressing TfR, such as cancer cells (Daniels *et al*., 2012). Thus, R‐Tf‐D‐LP4 targets HK preferably in cancer cells.

As expected from VDAC1‐based peptides that interact with HK, Bcl‐2, and Bcl‐xL and preventing their anti‐apoptotic activities (Abu‐Hamad *et al*., 2008; Shoshan‐Barmatz *et al*., 2009; Zaid *et al*., 2005), cell treated with the peptide underwent massive apoptosis, both in culture and in tumors (Figs 5 and 7, and S2). The peptide induced mitochondria‐mediated apoptosis, as reflected in the release of Cyto c, and the presence of apoptotic cells. As found for apoptosis inducers, such as STS, As_2_O_3_, and selenite (Weisthal *et al*., 2014), the peptide also disrupted [Ca^2+^]i homeostasis, increasing [Ca^2+^]i levels (Fig. 4). Furthermore, in tumors treated with the peptide, massive cell death was observed using TUNEL staining, and the levels of pro‐apoptotic proteins, Cyto *c*, p53, AIF, and caspases were highly elevated (Fig. 7). Hence, the dramatic effect of the peptide on tumor growth can be attributed to its action on two important cancer hallmarks, namely cancer cell energy status and induction of apoptotic cell death.

### 4.3 VDAC1‐based peptides similarly influence tumors derived from three different cancers by reprograming cell metabolism, inhibiting tumor growth, inducing cell death, and eliminating CSCs

In a recent study (Shteinfer‐Kuzmine *et al*., 2017), we demonstrated that in U‐87MG cell‐derived tumors, VDAC1‐based peptides perturbed cell energy homeostasis, inhibited tumor growth, induced apoptosis, and eliminated CSCs. Here, we demonstrate that the R‐Tf‐D‐LP4 peptide similarly affects tumors derived from the U‐87MG glioblastoma, A549 lung cancer, and the MDA‐MB‐132‐triple‐negative breast cancer cell lines. Indeed, in three different tumors, the R‐Tf‐D‐LP4 peptide similarly inhibited tumor growth (~80%) by inhibiting cell proliferation, as revealed by decreased Ki‐67 staining (Fig. 6F–H). This inhibition may result from the peptide impairing cancer cell energy and metabolism homeostasis (Fig. 8). Strikingly, the expression levels of energy‐related enzymes, such as certain enzymes associated with glycolysis (HK‐I, Glut‐1, and GAPDH), Krebs cycle activity [citrate synthase (CS)], and oxidative phosphorylation (complex IVc and ATP synthase 5a subunits), were downregulated, as revealed by IHC and qPCR (Fig. 8). These results suggest that in the residual peptide‐TTs, the reprogramed metabolism of the cancer cell was reversed upon interruption the interaction of VDAC1 with energy production‐associated enzymes, such as HK.

As demonstrated, VDAC1‐based peptides activate apoptosis in cells in culture (Figs 5 and S2) and in tumors (Fig. 7A–C). R‐Tf‐D‐LP4 induced massive apoptotic cell death in the three types of tumors tested. The intense apoptosis revealed by TUNEL staining, reflecting DNA degradation, can be explained by the multiple actions of the peptide, specifically antagonizing the anti‐apoptotic effect of anti‐apoptotic proteins, and inducing overexpression of key proteins in apoptosis, including caspases 3 and 8, p53, Cyto *c*, AIF, and SMAC/Diablo (Fig. 7D–I).

Finally, a large number of anticancer chemotherapeutic agents exert their therapeutic actions by inducing apoptosis of malignant cells (Costantini *et al*., 2000; Hail, 2005; Kaufmann and Vaux, 2003), mainly by activating the Cyto *c*/caspase‐9 pathway. However, they do not act on CSCs, which are resistant to chemo‐ and radiotherapies (Chen *et al*., 2012; Visvader and Lindeman, 2012). As shown in our previous study of GBM (Arif *et al*., 2017; Shteinfer‐Kuzmine *et al*., 2017), the R‐Tf‐D‐LP4 peptide eliminated CSCs in tumors derived from glioblastoma, lung, and breast cancers (Fig. 9).

The effect of the peptide on CSCs was also reflected in the inhibition of spheroid/aggregate formation (Fig. 9). Cell spheroid/tumorsphere‐forming capacity was used for evaluating the presence of CSCs (Valent *et al*., 2012; Weiswald *et al*., 2015). This effect may result from the peptide inhibiting CSC proliferation or/and inducing cell death.

The results reported here show that tumors derived from three different cell lines, despite carrying different mutations, responded similarly to the peptide. MDA‐MB‐231 (but not U‐87MG or A549) cells express mutated p53 and do not express the estrogen receptor (ER). At the same time, A549 cells express low levels of the pro‐apoptotic protein Bax and mutated KRAS, LKB1, and CDNKA, while U‐87MG cells express mutated PTEN. Thus, the anticancer activity of VDAC1‐based peptides is achieved regardless of cancer origin and mutational status and, most importantly, also involves the targeting of CSCs, known to be resistant to chemo‐ and radiotherapies.

## 5 Conclusions

To conclude, cell‐penetrating VDAC1‐based peptides were developed. The selected peptides possess multiple effects on cancer cells, including disruption of cell energy and metabolism homeostasis, and interference with the activity of anti‐apoptotic proteins, leading to apoptotic cell death with perceived specificity toward the cancerous cells.

In mouse models for GBM, and lung and breast cancers, the R‐Tf‐D‐LP4 peptide simultaneously attacked several cancer hallmarks, causing impairment of energy and metabolic homeostasis, including reduction in metabolic enzymes expression, inhibition of tumor growth, induction of apoptosis, and overexpression of pro‐apoptotic proteins. These manifold effects explain the prompt effect of the peptide on cancer cells. Moreover, the mode of action of the R‐Tf‐D‐LP4 peptide explains why it is not directed to a specific cancer type but rather acts on a variety of cancers, regardless of the mutations involved or the survival mechanisms acquired. These features of the protease‐protected, cell‐penetrating peptides point to their great therapeutic potential in various cancers, including those chemoresistant cancers for which traditional therapies are ineffective.

## Authors contributions

AS‐K, ZA, TA, and AZ performed the research and analyzed the data, and VS‐B assessed the results and wrote the manuscript.

## Supporting information


**Fig. S1.** Cell death‐inducing activity of various VDAC1‐based peptides
**Fig. S2.** Tf‐D‐LP4 and R‐Tf‐D‐LP4 induce apoptotic cell death
**Fig. S3.** Comparison between R‐Tf‐D‐LP4 and Tf‐D‐LP4 peptide treatments
**Table S1**. Amino acid sequences, MS/MS data, and analytical data for the peptides used in this study
**Table S2.** Antibodies used in this study
**Table S3.** Real‐time PCR primers used in this study
