# Supplementary material for: Selective induction of cancer cell death by VDAC1‐based peptides and their potential use in cancer therapy
Source: Mol Oncol. 2018 May 19;12(7):1077–103. doi: 10.1002/1878-0261.12313 (PMC6026870; doi:10.1002/1878-0261.12313)
Supplement: Supplementary file 1 — Fig. S1. Cell death‐inducing activity of various VDAC1‐based peptides Fig. S2. Tf‐D‐LP4 and R‐Tf‐D‐LP4 induce apoptotic cell death Fig. S3. Comparison between R‐Tf‐D‐LP4 and Tf‐D‐LP4 peptide treatments Table S1. Amino acid sequences, MS/MS data, and analytical data for the peptides used in this study Table S2. Antibodies used in this study Table S3. Real‐time PCR primers used in this study [file MOL2-12-1077-s001.docx]

**
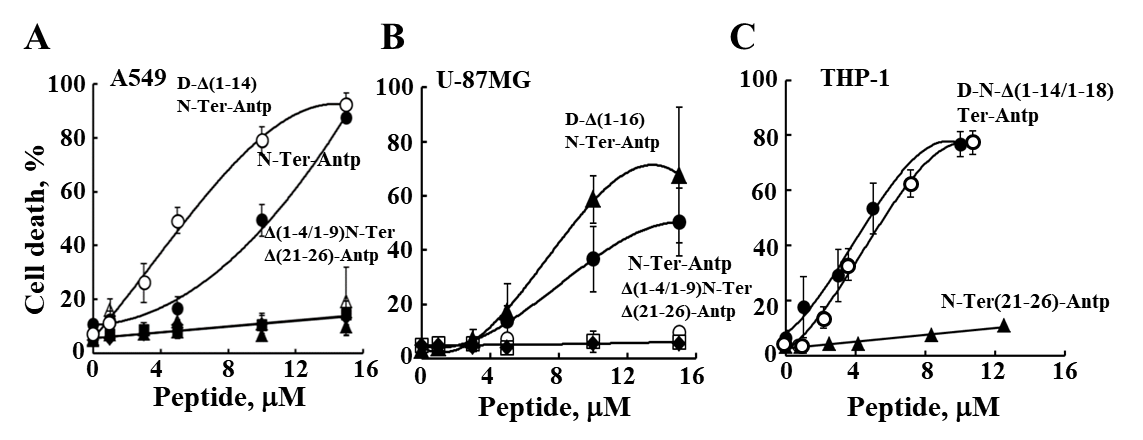
Supporting Information**

**Fig. S1. Cell death-inducing activity of various VDAC1-based peptides**

**A-C.** Versions of the N-terminal peptide assayed for cell death by PI staining and flow cytometry. **A**. A549 cells were incubated for 6 h with the indicated concentrations of the D-Δ(1-14)N-Ter-Antp (○), (1-26)N-Ter-Antp (●), Δ(21-26)N-Ter-Antp (▲), Δ(1-4,21-26)N-Ter-Antp (■), Δ(1-9,21-26)N-Ter-Antp (♦) or Antp (Δ) peptides and assayed for cell death. **B.** U-87MG cells were incubated for 5 h with the indicated concentrations of the (1-26)N-Ter-Antp (●), D-Δ(1-16)N-Ter-Antp (▲), Δ(21-26)N-Ter-Antp (○), Δ(1-4, 21-26)N-Ter-Antp (□) or Δ(1-9, 21-26)N-Ter-Antp (◊) peptides and assayed for cell death. **C**. THP-1 cells were incubated for 90 min with the indicated concentrations of the D-Δ(1-18)N-Ter-Antp (●), D-Δ(1-14)N-Ter-Antp (○) or Δ(21-26)N-Ter-Antp (▲) peptides and cell death was assayed.

**B**


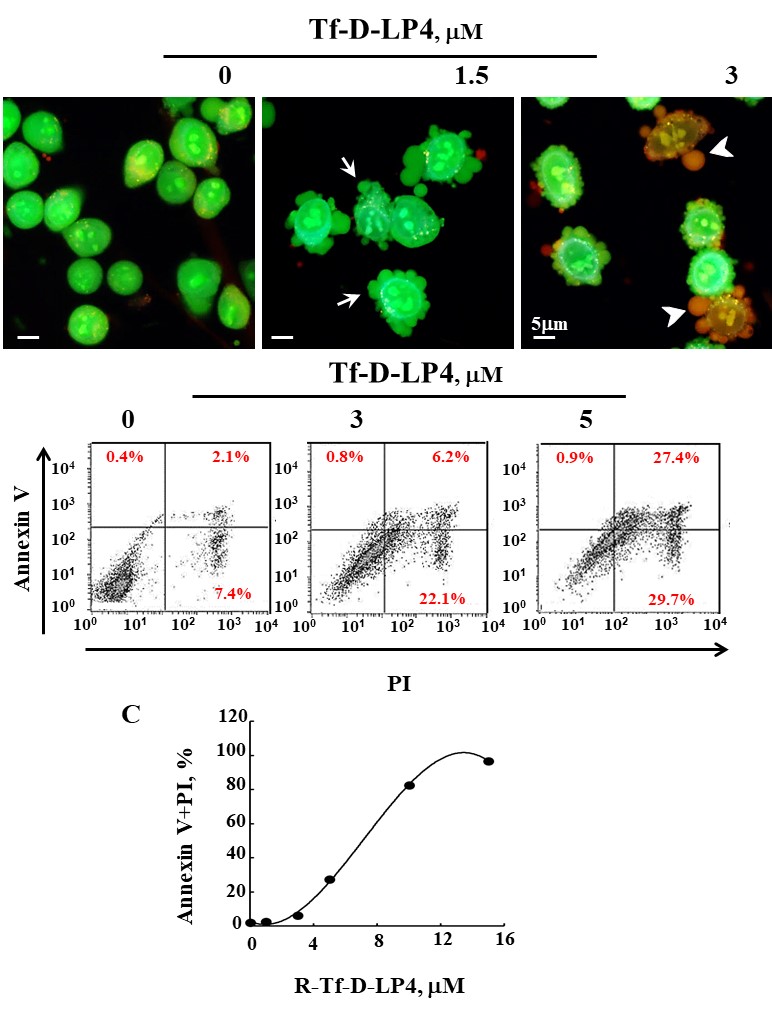


**A**

**C**

**Fig. S2. Tf-D-LP4 and R-Tf-D-LP4 induce apoptotic cell death**

**A.** HeLa cells were treated with the indicated concentrations of Tf-D-LP4 for 3 h and then stained with acridine orange and ethidium bromide (100 µg/ml). Arrows and arrowheads indicate cells with membrane blebbing (early apoptotic state) and cell is late apoptotic states, respectively. **B,C**. MDA-MB-231 cells were incubated for 3 h with DMSO (0.15%) or with the indicated concentrations of R-Tf-D-LP4 peptide in serum-free growth medium at 37^o^C and then subjected to FITC-Annexin V/PI staining and flow cytometry analysis. Representative FACS analysis of DMSO- (control) or R-Tf-D-LP4 (3, 5μM)-treated MDA-MB-231 cells (**B)** and quantitative analysis (**C)** are shown.

**
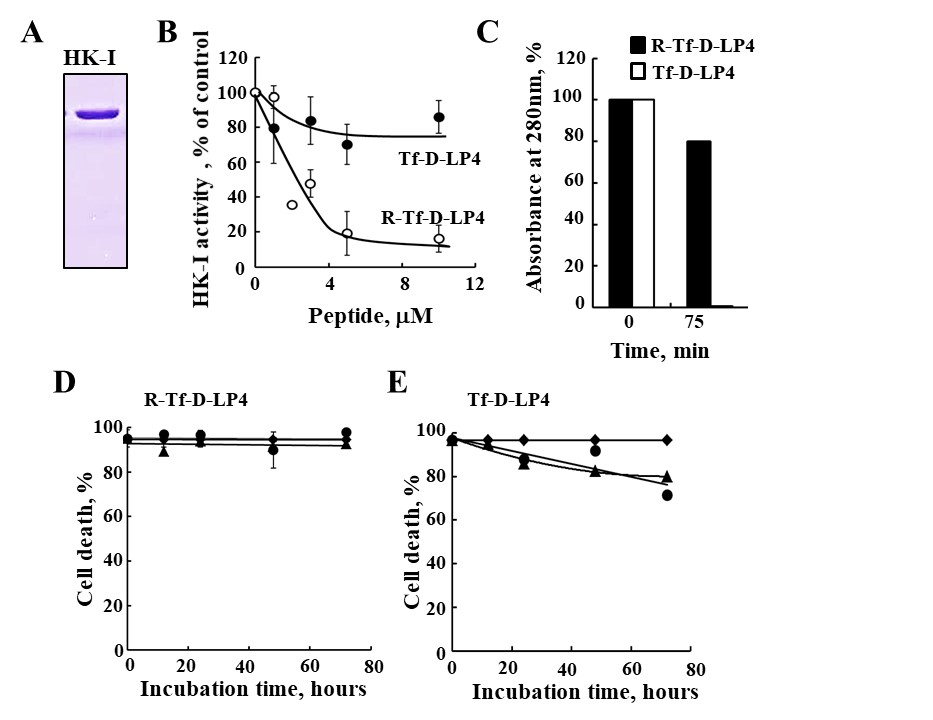
**

**Fig. S3. Comparison between R-Tf-D-LP4 and Tf-D-LP4 peptide treatments**

**A.** Purified HK-I. **B**. R-Tf-D-LP4 but not Tf-D-LP4 inhibits HK-I activity. Purified HK-I (13 μg/ml) was pre-incubated with the indicated concentrations of Tf-D-LP4 (●) or R-Tf-D-LP4 (○) and assayed for HK activity as described in Materials and Methods. Change in absorbance at 340 nm (NADH production) was spectrophotometrically measured following addition of 1 mM ATP, and 0.05 unit/ml glucose-6-phosphate dehydrogenase. **C.** Tf-D-LP4 and R-Tf-D-LP4 solubility. The peptides were dissolved to a final concentration of 5 mM in 5% DMSO and diluted to 1.1 mM in HBSS. Peptide absorbance in the supernatant was measured at 280 nm following centrifugation at 12,000g before and after 75 min incubation at 24^o^C. **D, E.** Tf-D-LP4 and R-Tf-D-LP4 (0.5 mM in 5% DMSO) were incubated at -20^o^C (**○**), 4^o^C (●) or 24^o^C (▲) and at the indicated time, aliquots (10 μM, final concentration) were assayed for cell death as described in Materials and Methods. Cell death was analyzed using PI and FACS analysis. In control cells, cell death was 3%, while peptide treatment led to levels of cell death over 90%.

1. **Supporting Tables**

**Supporting Table S1. Amino acid sequences, MS/MS data, and analytical data for the peptides used in this study**

The bold letters indicate the cell-penetrating peptide sequence while the underlined sequences represent amino acids involved in the tryptophan zipper (hairpin formation).

| **Peptide** | **Sequence** | **No. of AA** | **Molecular Mass, Da** | **Calculated ^a^** [**molar extinction coefficient**](https://en.wikipedia.org/wiki/Molar_absorptivity)**, M^-1^** | **Purity**  **%** |
| --- | --- | --- | --- | --- | --- |
| **Antp-LP4** | **RQIKIWFQNRRMKWKK-**SWTWE-199-KKLETAVNLAWTAGNSN-216-KWTWK | 43 | 5465 | 38500 | 89.02 |
| **D-Antp- LP4** | **RQIKIWFQNRRMKWKK-**SWTWE-199-KKLETAVNLAWTAGNSN-216-KWTWK | 43 | 5465 | 38500 | 85.53 |
| **Tf-LP4** | **HAIYPRH**SWTWE-199-KKLETA VNLAWTAGNSN-216-KWTWK | 36 | 4111 | 28990 | 92.22 |
| **Tf-D-LP4** | **HAIYPRH**SWTWE-199-KKLETA VNLAWTAGNSN-216-KWTWK | 34 | 4111 | 28990 | 95.94 |
| **R-Tf-D-LP4** | KWTWK-216-NSNGATWALNVATELKK-199-EWTWS**HRPYIAH** | 34 | 4111 | 28990 | 95.92 |
| **N-Ter-Antp** | 1-MAVPPTYADLGKSARDVFTKGYGFGL-26-**RQIKIWFQNRRMKWKK** | 42 | 4990 | 13980 | 97.72 |
| **Tf-∆(1-14)N-Ter** | 15-**RDVFTKGYGFGL**-26-HAIYPRH | 19 | 2235 | 2980 | 96.58 |
| **∆(1-14)N-Ter-Tf** | HAIYPRH**-**15-**RDVFTKGYGFGL**-26 | 19 | 2235 | 2980 | 95.75 |
| **D-Δ(1-14)N-Ter-Antp** | 15-RDVFTKGYGFGL-26-**RQIKIWFQNRRMKWKK** | 28 | 3588 | 12490 | 95.79 |
| **N-Ter∆21-26-Antp** | 1-**MAVPPTYADLGKSARDVFTK**-20-RQIKIWFQNRRMKWKK | 36 | 4396 | 12490 | 87.92 |
| **∆(1-4)N-Ter∆21-26-Antp** | 5-**TYADLGKSARDVFTK**-20-RQIKIWFQNRRMKWKK | 31 | 3901 | 12490 | 87.39 |
| **∆(1-9)N-Ter∆21-26-Antp** | 10-**LGKSARDVFTK**-20-RQIKIWFQNRRMKWKK | 27 | 3450 | 11000 | 85.18 |
| **D-∆(1-16)N-Ter-Antp** | 17-**VFTKGYGFGL**-26-RQIKIWFQNRRMKWKK | 26 | 3317 | 12490 | 95.17 |
| **D-∆1-18N-Ter-Antp** | 19-**FTKGYGFGL**-26-RQIKIWFQNRRMKWKK | 25 | 3218 | 12490 | 98.21 |

1. Calculated based on amino acid composition using the following link:

<http://www.biomol.net/en/tools/proteinextinction.htm>

**Supplementary Table S2. Antibodies used in this study**

Antibodies against the indicated protein, their catalogue number, source and the dilutions used in IHC and immunoblot (WB) experiments are presented. IF, immunofluorescence.

| **Antibody** | **Source and Cat. No.** | **Dilution** | |
| --- | --- | --- | --- |
|  |  | **IHC** | **WB** |
| Mouse monoclonal anti-actin | Millipore, Billerica, MA, MAB1501 | - | 1:40000 |
| Mouse monoclonal anti-ATP5a | Abcam, Cambridge, UK, ab14748 | 1:300 | - |
| Rabbit polyclonal anti-AIF | Abcam, Cambridge, UK, ab32516 | 1:200 | - |
| Rabbit polyclonal anti-CD44 | Abcam, Cambridge, UK, ab157107 | 1:400 | - |
| Rabbit polyclonal anti-citrate synthase | Abcam, Cambridge, UK ab96600 | 1:200 | - |
| Mouse monoclonal anti-cytochrome *c* | BD Bioscience, San Jose, CA, 556433 | 1:400 | 1:2000 |
| Rabbit monoclonal anti-cytochrome c oxidase subunit VIc | Abcam, Cambridge, UK, ab150422 | 1:200 |  |
| Mouse monoclonal anti-GAPDH | Abcam, Cambridge, UK, ab9484 | 1: 200 | 1:1000 |
| Rabbit monoclonal anti-Glut1 | Abcam, Cambridge, UK ab40084 | 1: 200 |  |
| Mouse monoclonal  anti-HK-I | Abcam, Cambridge, UK ab105213 | 1:500  1:250 IF | 1:2000 |
| Rabbit polyclonal anti-HK II | Abcam, Cambridge, UK ab3279 | - | 1:200 |
| Rabbit monoclonal anti-Ki67 | Thermo Scientific, NY RM-9106-s1 | 1:100 | - |
| Rabbit polyclonal anti-Klf4 | IMGENX Littleton, USA, IMG-6081-A | 1:200 | 1:1000 |
| Rabbit polyclonal anti-Nestin | Millipore, Billerica, MA, MAB353 | - | 1:25000 |
| Mouse monoclonal anti-P53 | Santa Cruz Biotechnology, Inc. , Dallas, TX, sc-126 | 1:400 | - |
| Goat polyclonal anti-SOX2 | Santa Cruz Biotechnology, Inc. Dallas, TX, sc-17320 | 1:200 | - |
| Rabbit polyclonal anti-S100b | Millipore, Billerica, MA, ABN59 | 1:200 | - |
| Rabbit polyclonal anti TfR | Abcam, Cambridge, UK, ab84036 | - | 1:2000 |
| Rabbit monoclonal anti-VDAC1 | Abcam, Cambridge, UK, ab15895 | 1:500 | 1:5000 |
| Goat anti-Rabbit | KPL, Gaithersburg, USA, 474-1506 | 1:250 | 1:15,000 |
| Goat anti-Mouse | Abcam, Cambridge, UK, ab97040 | 1:250 | 1:10,000 |
| Donkey anti-Goat | Abcam, Cambridge, UK, ab97120 | 1:250 | 1:20,000 |
| Donkey anti-Mouse (Alexa Fluor 488) | Abcam, Cambridge, UK, ab150109 | 1:250 IF |  |

**Supporting Table S3. Real-time PCR primers used in this study**

Names of the genes examined, and the forward and reverse sequences of the primers used for q-PCR are indicated.

| **Gene** | **Primers** |
| --- | --- |
| *ß-Actin* | Forward 5’-ACTCTTCCAGCCTTCCTTCC-3’  Reverse 5’- TGTTGGCGTACAGGTCTTTG-3’ |
| *AIF* | Forward 5'-AAGCACGCTCTAACATCTGG-3'  Reverse 5'-TTCTCCAGCCAATCTTCCAC-3' |
| *ATP Synthase 5a* | Forward 5'-TCAGTCTACGCCGCACTTAC-3’  Reverse 5'-GACATCTCAGCAGTCCCACA-3’ |
| *Citrate Synthase* | Forward 5’- AGGAACAGGTATCTTGGCTCT-3’  Reverse 5’- GGGGTGTAGATTGGTGGGAA-3’ |
| *Cytochrome c* | Forward 5′-TTTGGATCCAATGGGTGATGTTGAG-3  Reverse 5'-TTGAATTCCTCATTAGTAGCTTTTTTGAG-3 |
| *caspase 8* | Forward 5′-GGAGCTGCTCTTCCGAATTA-3'  Reverse 5'-GCAGGTTCATGTCATCATCC-3' |
| *Caspase 3* | Forward 5'-TTCAGAGGGGATCGTTGTAGAAGTC-3′  Reverse 5′-CAAGCTTGTCGGCATACTGTTTCAG-3′ |
| *GAPDH* | Forward 5’- TGGAAGGACTCATGACCACA-3’  Reverse 5’- ATGATGTTCTGGAGAGCCCC-3’ |
| *GLUT1* | Forward 5’- GGCCATCTTTTCTGTTGGGG-3’  Reverse 5’- TCAGCATTGAATTCCGCCG-3’ |
| *HK-I* | Forward 5’-GTCTCAGTCCAGCACGTTTG-3’  Reverse 5’- GAAACGCCGGGAATACTGTG-3’ |
| *Ki-67* | Forward 5’- CTTTGGGTGCGACTTGACG-3’  Reverse 5’-GTCGACCCCGCTCCTTTT-3’ |
| *LDH-A* | Forward 5’-GCAGGTGGTTGAGAGTGCTT-3’  Reverse 5’- GCACCCGCCTAAGATTCTTC-3’ |
| *p53* | Forward 5′-AGGTTGGCTCTGACTGTACC-3′  Reverse 5′-AAAGCTGTTCCGTCCCAGTA-3 |
| *Smac/Diablo* | Forward 5’-CTGACTTCTACTTCCAGGCTGTT-3'  Reverse 5’- GCTCCTATGATCACCTGCCA-3' |
| *VDAC1* | Forward 5’- AATGACGGGACAGAGTTTGG-3’  Reverse 5’- AGCGCGTGTTACTGTTTCCT-3’ |
